# Gene duplication is associated with gene diversification and potential neofunctionalization in lung cancer evolution

**DOI:** 10.1101/gr.278663.123

**Published:** 2026-03

**Authors:** Paul Ashford, Alexander M. Frankell, Zofia Piszka, Camilla S.M. Pang, Mahnaz Abbasian, Maise Al Bakir, Mariam Jamal-Hanjani, Nicholas McGranahan, Charles Swanton, Christine A. Orengo

**Affiliations:** 1 Institute of Structural and Molecular Biology, University College London, London WC1E 6BT, United Kingdom;; 2 Cancer Evolution and Genome Instability Laboratory, The Francis Crick Institute, London NW1 1AT, United Kingdom;; 3 Cancer Research UK Lung Cancer Centre of Excellence, University College London Cancer Institute, London WC1E 6DD, United Kingdom;; 4 University College London Cancer Institute, University College London, London WC1E 6DD, United Kingdom;; 5 Cancer Metastasis Laboratory, University College London Cancer Institute, London WC1E 6DD, United Kingdom;; 6 Department of Oncology, University College London Hospitals, London NW1 2BU, United Kingdom;; 7 Cancer Genome Evolution Research Group, Cancer Research UK Lung Cancer Centre of Excellence, University College London Cancer Institute, London WC1E 6DD, United Kingdom

## Abstract

Tumors evolve through a process of selection on somatic mutations, driving cell division and tissue growth through aberrations in cell-cycle control. In non-small-cell lung cancer (NSCLC), genome instability occurs early in tumor growth, resulting in pronounced intratumor heterogeneity, including changes in gene copy number, and whole-genome doubling (WGD) in ∼75% of tumors. Gene duplication, genetic drift, and selection mediate functional diversification during evolution. In this study, we seek to identify the diversification and potential gene neofunctionalization of lung tumors in the TRACERx cohort. We develop a novel computational protocol to identify preduplication and postduplication mutations predicted to affect protein function. Mutations are analyzed using paralogs grouped into functional families with highly similar functions, identifying 355 functional impact events (FIEs) through their proximity and clustering near to functional sites. The use of functional family paralogs to map mutations to protein structures from the PDB helps predict putative rare driver events in lung tumors. By extending the analysis with high-quality structural models from AlphaFold using The Encyclopedia of Domains (TED), we find a significant increase in the diversity of both genes and functional families with postduplication FIEs in lung adenocarcinomas, including some metabolic enzymes with the potential to be neofunctional. The postduplication diversification of driver genes and functions may indicate selection for somatic copy number changes in lung tumors and an increased scope for tumor adaptations.

Cancers result from the evolutionary processes of selection on somatic mutations, driving tumor growth through aberrant regulation of the cell cycle. Somatic mutations caused by multiple endogenous and exogenous factors are mostly neutral passengers, with only a few drivers of clonal expansion under strong positive selection. Cancer genomic studies have sought to catalog driver events occurring in hundreds of cancer genes across different tumor types (Martincorena and Campbell 2015; Chung et al. 2016; Tokheim and Karchin 2019; Dietlein et al. 2020; Kumar et al. 2020; The ICGC/TCGA Pan-Cancer Analysis of Whole Genomes Consortium 2020). Although this dichotomous view of mutation effect has proven useful, the expansion of cancer genomic data has aided in the development of more nuanced models. Some passengers may be weak drivers that contribute to tumor fitness in combination with other mutations (Cannataro and Townsend 2018; Kumar et al. 2020; Ostroverkhova et al. 2023). Drivers can exhibit patterns of co-occurrence or mutual exclusivity, as observed between *EGFR* and *KRAS* in lung adenocarcinomas (LUADs), and such patterns may be common across all cancer types (Sinkala 2023). Pairwise epistasis may link one driver to a higher likelihood of another occurring through increased selection, such as between *TP53* or *LRP1B* drivers and *KRAS* in lung cancers (Alfaro-Murillo and Townsend 2023). These conditional positive or negative selection pressures between gene pairs may occur in up to half of all drivers in some tumors (Iranzo et al. 2022). Furthermore, higher-order epistasis may play a small yet significant role in tumor evolution and complicate predictions from models based solely on pairwise associations (Sailer and Harms 2017; Weinreich et al. 2018; Alfaro-Murillo and Townsend 2023). The tumor microenvironment influences the overall magnitude and direction of selective pressure through changes in the extracellular matrix, nutrient availability, immune system cells, cytokines, and T cell regulators (Anari et al. 2018; Mansouri et al. 2022; Nakayama et al. 2022; Otohinoyi et al. 2022).

In non-small-cell lung cancer (NSCLC), pronounced intratumor heterogeneity (ITH) arises from genomic instability processes that occur early during tumor growth, which increases the overall mutation rate and leads to greater genetic diversity (De Bruin et al. 2014; Jamal-Hanjani et al. 2017; Zhang et al. 2017). Tumors with higher levels of ITH are associated with a poorer prognosis (Jamal-Hanjani et al. 2017). Through increased genetic diversity, tumors with higher ITH are more likely to possess variants with beneficial functions under different conditions, leading to subclonal expansions (López et al. 2020). The complex and dynamic selection pressures of the tumor microenvironment on this heterogeneous mutation landscape can lead to the development of treatment-resistant subclones and influence the choice of therapy (Mumenthaler et al. 2015; Zhang et al. 2017; Fisk et al. 2022). Treatment regimens further influence clonal selection and complicate decisions regarding appropriate therapy choice and timing (Black and McGranahan 2021; Fisk et al. 2022).

Gene duplication during species evolution is an important mechanism that permits genetic diversity and the development of new functions through drift and selection (Ohno 1970; Taylor and Raes 2004). Duplicated genes are usually lost over short evolutionary timescales, but they can be retained through three primary mechanisms: conservation of wild-type function, in which duplicates alter gene dosage or provide redundancy; subfunctionalization, in which ancestral function is partitioned across copies; and neofunctionalization, in which duplicates evolve new functions (Taylor and Raes 2004; Hahn 2009; Kondrashov 2012; Kuzmin et al. 2022). Genome sequencing has supported the view that whole-genome duplication (WGD) events frequently occur during evolution across all domains of life, with many plant species retaining polyploid genomes (Van de Peer et al. 2009; Kondrashov 2012; Wang et al. 2012). Although increased ploidy following WGD incurs fitness costs to an organism, it can confer advantages during times of environmental change and stress, including increased adaptability and robustness of gene regulatory networks (Ebadi et al. 2023) by supporting the divergence and adaptation of species lineages to distinct niches (Robertson et al. 2017; Kuzmin et al. 2022), or by the development of new protein functions through neofunctionalization. In this study, putative neofunctional events in lung tumors are explored with particular reference to the “innovation–amplification–divergence” model of neofunctionalization (Näsvall et al. 2012), in which a pre-existing, weak, secondary gene function (“innovation”) confers advantage owing to changes in the environment, leading to selection for increased gene copy (“amplification”) number and subsequent divergence of function (Hahn 2009).

Approximately 75% of lung cancers have WGD events during their evolutionary history (Jamal-Hanjani et al. 2017). Furthermore, changes in gene copy number owing to aneuploidy, in which chromosomes or chromosomal regions are gained or lost, represent an important class of driver events in many cancer types (Zack et al. 2013; The ICGC/TCGA Pan-Cancer Analysis of Whole Genomes Consortium 2020; Watkins et al. 2020). Tumors with WGD have more diverse patterns of aneuploidy because of the resilience provided by extra functional gene copies (Klockner and Campbell 2024) and may also provide a buffer for the continuous accumulation of deleterious mutations in essential genes (López et al. 2020). Conceivably, tumor WGD and gene duplication events may also broaden the cellular evolutionary potential of cancer cells, permitting divergence in duplicated genes and functional modifications that, through benefits to the tumor, are under positive selection, leading to neofunctionalization.

In this study, we used computational strategies to identify mutations that could affect protein function through their proximity to functional sites. These mutations may be inactivating (causing loss of function), activating, latent (enabling another distinct driver mutation) (Nussinov and Tsai 2015), or, in rare cases, neofunctional, in which the protein function differs from the wild type and benefits the tumor. Many computational tools have been developed to predict cancer driver genes and mutations using data sets from tumor sequencing projects, such as The Cancer Genome Atlas (TCGA) (Hutter and Zenklusen 2018; The ICGC/TCGA Pan-Cancer Analysis of Whole Genomes Consortium 2020). These tools employ a variety of approaches, including analysis of mutations using gene sequences, protein sequences, and protein structures (Porta-Pardo et al. 2017; Bailey et al. 2018; Muiños et al. 2021; Ostroverkhova et al. 2023). We developed a protocol based on our previous work that showed how protein domain families in the CATH protein structure classification database (Sillitoe et al. 2015) can help group paralogous domains from different human proteins into functional families, in which relatives share highly similar structures and functions (Sillitoe et al. 2015; Das et al. 2015b). These families can be used to group mutations observed in paralogs to identify common effects on protein function (Ashford et al. 2019). In addition, using protein structure provides insights into how mutations affect protein function through effects at key functional sites. We hypothesized that protein domains may contain “tunable sites,” near to functional sites, which may be similarly altered in multiple tumor types and can be identified by significant clustering of mutations in three dimensions. We defined mutations that modified protein function via effects at or near protein functional sites as functional impact events (FIEs).

This study developed a novel protocol (“FunVar”) to analyze mutations identified in lung tumors from TRAcking Cancer Evolution through therapy (Rx) (TRACERx) and predict FIEs. By classifying FIEs by mutation timing relative to gene duplications, we aimed to discern changes in gene and functional diversity during lung tumor evolution, including cases in which gene duplication may have permitted neofunctionalization.

## Results

### Development of FunVar to predict FIEs

**Figure 1.**
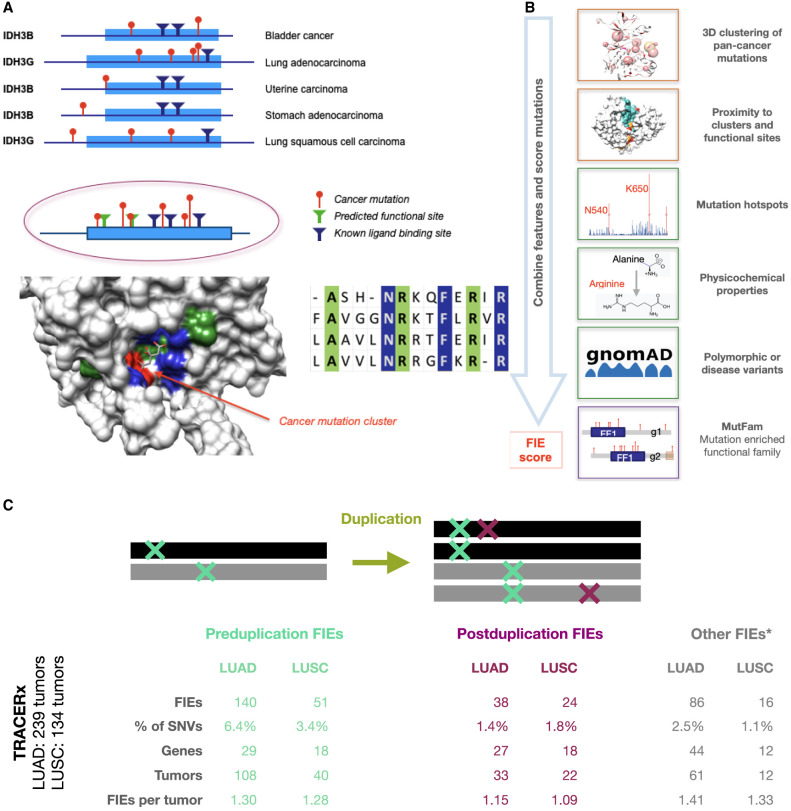
FunVar protocol identifies FIEs in lung tumor evolution. (*A*) FunVar protocol. Mutations (red) from different tumor types and gene paralogs were grouped when they shared protein domains predicted to have equivalent functions because they belong to the same functional family in the CATH database (Sillitoe et al. 2015; Das et al. 2015b). These mutations can be mapped to a single 3D structural representative. Known functional sites from paralogs (blue), and likely functional sites predicted owing to high conservation within the CATH functional families (green), are mapped in an identical manner. Significant mutation clusters near functional sites were termed “tunable sites,” highlighting commonalities between mutations from different cancer types and paralogs via their impacts on specific protein functions and aiding the detection of rare events. (*B*) FunVar scoring. TRACERx lung mutations were tested for occurrence in tunable sites and given functional impact event (FIE) scores using a simple heuristic based on mutation properties. (*C*) FIEs identified pre- and postduplication for LUAD and LUSC. Number of FIEs, FIEs as percentage of SNVs (missense and synonymous), number of distinct genes containing FIEs; number of tumors with FIEs, and average FIEs per tumor. Other FIEs occur in regions with no gene duplication, although a minority of these occur in areas of monoallelic duplication when the timing of the mutation relative to the duplication may be unknown.

We developed a computational protocol, FunVar, to predict mutations likely to impact protein function via action at functional sites, which we termed functional impact events (FIEs) (Fig. 1A,B; Methods). This protocol first used TCGA pancancer nonsynonymous single-nucleotide variants (SNVs) to generate a comprehensive set of mutation clusters on protein domain structures and then labeled clusters within 5 Å (5 Å = 5 × 10^−10^ m) of a functional site as “tunable sites.” These tunable sites were used to identify FIEs in lung tumors, identify both known and predicted novel driver mutations, and provide a basis for identifying putative novel neofunctional events (Fig. 1). Other methods have used 3D clustering of mutations (Porta-Pardo and Godzik 2014; Niu et al. 2016; Tokheim et al. 2016; Porta-Pardo et al. 2017; Muiños et al. 2021) to identify drivers; FunVar employs a novel approach that first identifies protein domains in which mutations occur and then collates these domains with paralogs that have the same function, as defined by CATH functional families (FunFams) (Das et al. 2021). We used this strategy as both the collation of mutations and the transfer of functional site annotations between paralogs are more accurate when using functional families than superfamilies, which may have become functionally diverse over time (Kryuchkova-Mostacci and Robinson-Rechavi 2016). The tunable sites are the specific protein residues and functional sites that are commonly targeted, with equivalent effects on protein function, in these sets of domain paralogs.

FunVar FIEs are a specific class of driver mutations focused on predicted impacts on functional sites, rather than general cancer driver predictions. Nonetheless, FunVar performance is comparable to that of contemporary cancer driver prediction tools that utilize protein structures. Benchmarking comparisons with HotSpot3D (Niu et al. 2016), HotMAPS (Tokheim et al. 2016), and 3dHotSpots (Gao et al. 2017), with reference to drivers from the COSMIC Cancer Mutation Census (Tate et al. 2019), are summarized in Supplemental Figure 13 (for additional information, see Supplemental Note 1; Supplemental Fig. 13). Cases in which known drivers were identified by other tools but not by FunVar (reflected in lower F1-scores; FunVar, 0.703, vs. other tools, 0.719–0.735) (Supplemental Fig. 13) occurred for two reasons. FunVar has strict data requirements that exclude mutations not assigned to a functional family with site annotations and a domain structure (62% of cases). Second, the protocol was designed to exclude mutations that were not part of a statistically significant mutation cluster or hotspot (35% of cases; significance level of 5%). The majority of cases in which mutations were not clustered occurred in tumor-suppressor genes (*TP53*, *PTEN*, and *VHL*) (Supplemental Note 1; Supplemental Fig. 14), which acquire loss-of-function variants across a larger proportion of sites and in fewer compact clusters than oncogenes (Martínez-Jiménez et al. 2020). FunVar also uniquely identified a set of drivers (see Discussion) (Supplemental Fig. 14; Supplemental Table 11).

The pancancer mutation clusters near functional sites were then used to identify FIEs from missense SNVs in the TRACERx NSCLC study, using tumors classified as either LUAD (n = 239 tumors) or lung squamous cell carcinoma (LUSC; n = 134 tumors). Missense mutations from TCGA-lung tumors were used to supplement the TRACERx analysis in some instances (TCGA-lung data set: LUAD, n = 387 tumors; LUSC, n = 342 tumors; see Methods) (Supplemental Table 1; Supplemental Fig. 10).

FIEs associated with gene duplication can be timed using their estimated mutation copy number as likely to have occurred either before or after the gene duplication event. Preduplication mutations were defined as those with at least two mutant copies in a genomic area where a gain has occurred; postduplication mutations, as those with a single copy, as previously described (see Methods) (Jamal-Hanjani et al. 2017; Gerstung et al. 2020). These timed FIEs (Fig. 1C) were then used to identify differences between preduplication and postduplication in terms of their distribution in genes and protein domains and their impacts on function.

### FIEs include both established drivers and predict novel events

FunVar analysis, scoring, and filtering of LUAD and LUSC tumors in TRACERx (n = 373 tumors) identified 355 FIEs in 224 tumors (Supplemental Table 2). In total, FIEs were found in 109 genes, of which 57% had enzyme functions (Supplemental Table 3). The FIE-containing protein domains belong to 95 functional families (Supplemental Tables 2, 4). There were fewer functional families than genes because FunVar groups domains in different gene paralogs that share a common function (via CATH, see Methods; note “paralog” refers to genes present in the human genome not to copies arising from duplication events such as WGD). Filtering steps removed FIEs identified in genes without significant expression in TRACERx lung tumors (LUAD, 3/80 genes; LUSC, 4/41 genes) or where the FunVar score was below the threshold (see Methods).

**Figure 2.**
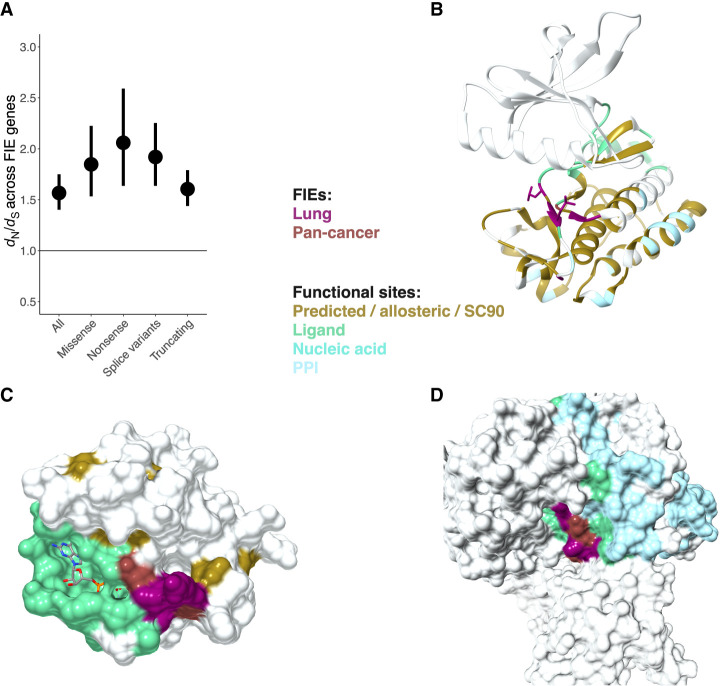
Lung FIE genes are under positive selection and include both known cancer genes and predicted novel drivers in paralogs that are also likely to affect protein function. (*A*) FIE genes (score ≥ 3) were under positive selection (*d*_N_/*d*_S_ > 1.5). (*B*) FIEs in ERBB tyrosine–protein kinase paralogs EGFR* (lung), ERBB4* (lung), and ERBB2* (pancancer). (*C*) Ras small monomeric GTPase with LUAD FIEs in genes *RRAS2* and *RIT* within the same FunFam. (*D*) Rac family small GTPase with LUSC FIEs in genes RAC1* and *RAC2* within the same FunFam. (*) Known cancer genes from CGC.

FIE genes showed signals of positive selection, assessed using *d*_N_/*d*_S_ (Fig. 2A; Supplemental Note 4), indicating significant enrichment in missense, nonsense, splice, and truncating mutations compared with synonymous variants. Pancancer FIEs showed positive cancer effect sizes (CESs) compared with dbSNP polymorphisms (Supplemental Figs. 15, 16; Supplemental Note 5; Supplemental Code 1). In addition, FIE genes were depleted in germline missense mutations (*P* = 0.002, Welch's *t*-test) compared with genes without FIEs, indicating negative selection for germline missense variants in FIE genes (Supplemental Fig. 1).

Figure 2, B through D, provide illustrative examples of FIEs in three functional families in which mutations affected the same functional site in multiple paralogs, including those in the known cancer genes *EGFR* and *RAC1*. Although this study focused on lung tumors, the FunVar method for identifying FIEs used a TCGA pancancer data set, which we refer to when helpful for providing supporting evidence or insights. For example, FIEs may be identified in specific paralogs in a functional family according to cancer type. This paralog specificity was observed in the kinase domain of the ERBB protein family, in which TRACERx lung FIEs only occurred in *EGFR*, whereas in bladder, uterine, and pancreatic cancers, the paralogs *ERBB2* and *ERBB4* were most commonly affected.

**Figure 3.**
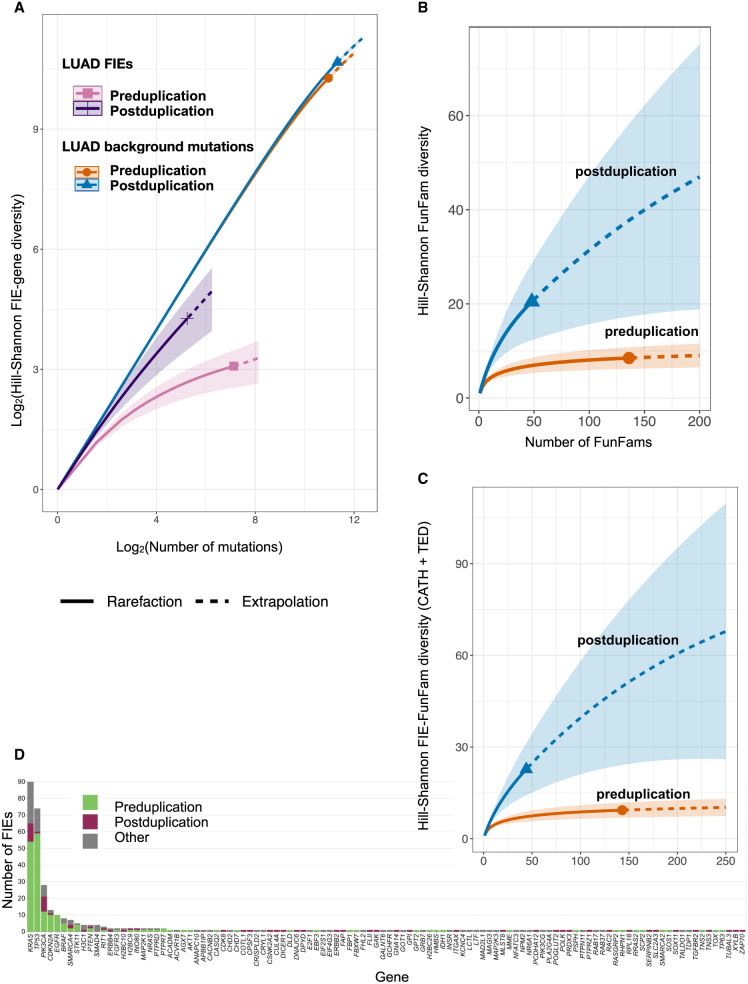
Hill–Shannon FIE-gene and functional family diversity in LUAD before and after duplication. (*A*) Hill–Shannon FIE-gene diversity significantly increased in LUAD tumors postduplication (95% CI, bootstrapped). Background-mutation-gene diversities did not show an equivalent postduplication increase (95% CI, bootstrapped). Log_2_–log_2_ plots compare pre- and postduplication FIE/mutation-gene diversity as a function of the number of FIEs/mutations, using rarefaction to estimate diversity at smaller sample sizes than observed (solid line) and extrapolation to estimate more than twice the observed number of FIEs/mutations sampled (dotted line). (*B*) Hill–Shannon FIE-FunFam diversity for FIEs derived using PDB structures. (*C*) Hill–Shannon FIE-FunFam diversity for FIEs derived using PDB structures and TED/AlphaFold models. (*D*) The long tail in the FIEs per gene distribution.

Overall, 264 TRACERx FIEs (74.4% of the FIEs identified) were found in 25 known cancer genes, with an average of more than 10 FIEs per gene. Only seven genes had FIEs identified in more than five tumors, all of which were known cancer genes: *TP53* (n = 74 FIEs), *PIK3CA* (28), *CDKN2A* (13), *KRAS* (90), *SMARCA4* (7), LUAD-specific *EGFR* (10), and *BRAF* (8). Using driver mutations classified with the TRACERx pipeline (Frankell et al. 2023), 254 FIEs (71.5%) were classed as TRACERx drivers and with similar frequency in LUAD and LUSC (about 1.3 FIEs per tumor). The remaining 91 FIEs occurred in noncancer genes (n = 84 genes in 69 tumors), corresponding to just greater than one FIE per gene. The tendency for the majority of FIEs to fall into only a few genes, with many genes containing only a single FIE, gave rise to a long-tailed distribution of FIEs per gene (Fig. 3; Supplemental Fig. 2). All FIEs, genes, and functional families are provided for NSCLC (Supplemental Table 2) and pancancer analysis (Supplemental Table 4).

### A majority of FIEs occur preduplication in a handful of established cancer genes

Over half of FIEs likely occurred as preduplication (early in tumor development; LUAD 53% and LUSC 56% of total FIEs) (Fig. 1C). In total, 88% of preduplication FIEs (n = 168 FIEs) occurred in known cancer genes, with the majority (n = 137 FIEs, 72% of preduplication FIEs) in *KRAS*, *TP53*, *PIK3CA*, *CDKN2A*, *EGFR*, and *BRAF*. Compared to timed synonymous SNVs, FIEs were significantly enriched preduplication compared with postduplication in both LUAD and LUSC (LUAD *P* < 0.001, LUSC *P* < 0.001; chi-squared), with a similar signal found in TCGA-lung (LUAD *P* = 0.034, LUSC P = 0.018; chi-squared).

Preduplication FIEs in LUAD include *EGFR* drivers L858R (n = 7 FIEs) and L861Q (n = 2 FIEs), which are known to promote proliferation through constitutive activation of intracellular kinase domains. Additional *EGFR* kinase sites were identified using LUAD tumors from TCGA-lung (L833 and S752, each with two FIEs). For these sites, FIEs at equivalent positions in paralogs *ERBB4* and *ERBB2* (Fig. 2B) provided additional evidence of positive selection of functional impact.

In the set of preduplication FIEs, 85% (n = 162 FIEs) were independently classified as driver mutations by TRACERx, with the remaining 15% representing putative novel drivers identified by FunVar. Preduplication FIE genes were enriched in cancer hallmark processes including regulation of cell development, signal transduction, cell-cycle processes, and regulation of growth (*P* < 5 × 10^−9^, GO:BP, g:Profiler) (Supplemental Table 9).

### Postduplication FIEs are distributed in a wider variety of genes, with less than half occurring in known cancer genes

Fewer FIEs were identified postduplication compared with preduplication (LUAD, 18.5%; LUSC, 30.8% of total FIEs), and less than half were in known cancer genes (46%, n = 35/76 postduplication FIEs). Although fewer postduplication FIEs were found in known cancer genes, there was no significant difference in the predicted pathogenicity scores compared with preduplication (CADD [Rentzsch et al. 2019], *P* = 0.6185; PolyPhen [Adzhubei et al. 2013], *P* = 0.155; SIFT [Vaser et al. 2016], *P* = 0.5695; Welch's *t*-test), and pathogenicity scores for FIEs were significantly higher than for non-FIE missense mutations. Postduplication FIE genes were enriched in processes including cell population proliferation, cellular component disassembly, metabolic processes, cell-cycle regulation, and apoptotic/cell-death processes (*P* < 4.2 × 10^−9^, GO:BP, g:Profiler) (Supplemental Table 9).

Only two genes, *KRAS* and *PIK3CA*, had postduplication FIEs in more than five tumors (n = 11 and 9 FIEs, respectively), and both of these were more commonly affected preduplication; thus, the major contribution to a “long-tail” in the FIEs per gene distribution is from increased diversity of FIE genes (“FIE-gene diversity”) postduplication (Supplemental Fig. 2). Compared with preduplication, these postduplication FIEs occur in fewer known cancer genes, resulting in 33 putative novel drivers.

We quantified FIE-gene and FIE-functional family diversity using Hill metrics, commonly used in ecology, to compare species diversity between different locations. We calculated Hill–Shannon diversity scores using a bootstrap subsampling (rarefication) and extrapolation method (iNEXT.3D) (Chao et al. 2021) to allow for meaningful comparisons of diversity scores when the sample sizes differed between groups, such as between the number of FIEs per gene or family identified pre- and postduplication. Here, Hill–Shannon diversity indices were calculated using either FIEs or background mutations (missense SNVs that were neither FIEs nor TRACERx drivers) per gene or functional family, comparing those found pre- and postduplication, and capture diversity by analyzing the distribution of FIE (or mutation) counts per gene (Fig. 3A) or functional family (Fig. 3B). Hill–Shannon metrics are useful for capturing diversity differences when there are both rare and common species (e.g., FIE genes) in the data set. Other metrics, such as Hill–Simpson, provide scores weighted toward the most common events, and species richness estimates the total number of species (i.e., genes or functional families; see Methods).

These Hill diversity calculations indicate that TRACERx FIEs in LUAD tumors have a significantly higher postduplication FIE-gene diversity (bootstrapped 95% confidence), with similar findings for functional family diversity (Fig. 3B); for LUSC, a similar trend was observed, but there were too few FIEs to ascertain significance at 95% confidence (Supplemental Fig. 3).

This increased postduplication FIE-gene and FIE-functional family diversity relative to background mutations provides evidence for the selection of gene duplication events. The increased copy number allows for functional variations in genes while preserving wild-type functions and can thus diversify functional adaptations in tumors.

As FIEs were identified using functional families with PDB structures, they represent a subset of the total possible FIEs that could be identified given increased structural coverage. To increase confidence that the observed increase in postduplication diversity reflected a process in tumor evolution and was not a result of the underlying data set distribution, we extended the diversity analysis to include AlphaFold (Varadi et al. 2022) predicted protein structure models obtained from The Encyclopedia of Domains (TED) (Lau et al. 2024). Using an additional 1040 high-quality models for functional families lacking PDB structures, we identified an additional 19 FIEs in TRACERx, including six postduplication in LUAD and two in LUSC (Supplemental Note 2; Supplemental Table 10). Hill–Shannon diversity was calculated for the pooled (i.e., PDB and TED-derived) FIEs in LUAD (Fig. 3C). These additional FIEs lend weight to the finding of significantly increased FIE-gene and FIE-functional family diversity in LUAD (95% confidence).

Increased diversity can also arise from genome instability processes independent of copy number changes; these processes increase the tumor mutation burden and thus the likelihood of novel driver mutations. Mutations arise through impairments in DNA-damage repair pathways (Wu et al. 2020), endogenous processes including APOBEC mutagenesis, and exogenous factors such as smoking (Alexandrov et al. 2020). In lung tumors, such processes can result in a wider range of subclonal driver events, as previously observed (Jamal-Hanjani et al. 2017; Frankell et al. 2023).

Separating the influences of gene duplication and mutation timing is difficult, and in TRACERx and other studies, mutation timing was defined with reference to gene duplication events (Jamal-Hanjani et al. 2017; Gerstung et al. 2020; Ramesh et al. 2022; Frankell et al. 2023).

We sought to address the potentially confounding influence of mutation timing on postduplication FIE-gene diversity by comparing clonal and subclonal FIEs from tumors with duplicated regions to those with none. Genes with subclonal FIEs were more diverse than those with clonal FIEs, irrespective of duplication status (Supplemental Fig. 4). There was also an indication that duplication increased the subclonal FIE-gene diversity in LUAD tumors. However, we could not ascertain the significance at the 95% CI from either PDB-derived or pooled PDB/TED FIEs. The wide 95% confidence intervals for both clonal and subclonal FIEs in regions without gene duplication reflect the small number of FIEs that were identified in these classes (clonal n = 13 and subclonal n = 17 FIEs). The sample size was insufficient to determine any trends in LUSC (Supplemental Fig. 4).

### Postduplication FIEs in diverse protein families are potentially neofunctional

Increased postduplication FIE-gene and FIE-functional family diversity indicates potential selection of gene duplication events as a way of enhancing tumor adaptability. In addition, it is possible that some FIEs (including those in known cancer genes) result in neofunctional changes. Here, neofunctionalization refers to the emergence of a tumor-beneficial protein function that is distinct from the wild-type function, such as mutations near ligand binding sites altering substrate specificity of an enzyme or favoring secondary existing functions of multifunctional proteins (“moonlighting” proteins) (Bergthorsson et al. 2007; Copley 2020). For example, we identified a postduplication *IDH1* FIE in a TCGA-LUAD tumor with an R132C mutation. This mutation has been shown to be a neofunctional driver in a subset of gliomas, resulting in a gain of enzyme function and production of the oncometabolite D-2-hydroxyglutarate (Dang et al. 2010). Although *IDH1* mutations are common in a subset of gliomas, they are infrequent in LUAD, occurring in <1% of tumors (Rodriguez et al. 2020), in agreement with previous observations in which known drivers frequently found in one cancer type occurred as rare drivers in another (Armenia et al. 2018; Cannataro and Townsend 2019).

To identify putative neofunctional events, we used additional evidence to supplement our FunVar protocol. Although FIEs are predicted to affect protein function owing to their proximity to functional sites, they do not directly indicate how functions are affected. These effects include inactivation (loss of function), activation, changes to biological pathways through modification of a protein–protein interface (PPI), and neofunctionalization. Therefore, we assessed each FIE using additional evidence to indicate its potential for neofunctionalization by (1) identifying whether the FIE gene was from a diverse CATH superfamily, (2) checking for paralogs with known moonlighting functions, (3) analyzing the FIE's context in the protein structure, and (4) making inferences based on literature support for the role of the gene in cancer (see Methods).

We identified 28 functional families in which one or more paralogs contained postduplication or subclonal FIEs, in which the expression of the mutation was confirmed in the TRACERx cohort by RNA-seq (Supplemental Table 5; Frankell et al. 2023). Positive CESs were observed in FIEs in a majority of these functional families (n = 24/28 families) in TCGA pancancer (Supplemental Fig. 17), with somewhat larger effects also observed in subsets of these families in the lung-specific cohorts TRACERx LUAD (Supplemental Fig. 18) and LUSC (Supplemental Fig. 19). For further information on CES analyses, see Supplemental Note 5 and Supplemental Code 1.

**Figure 4.**
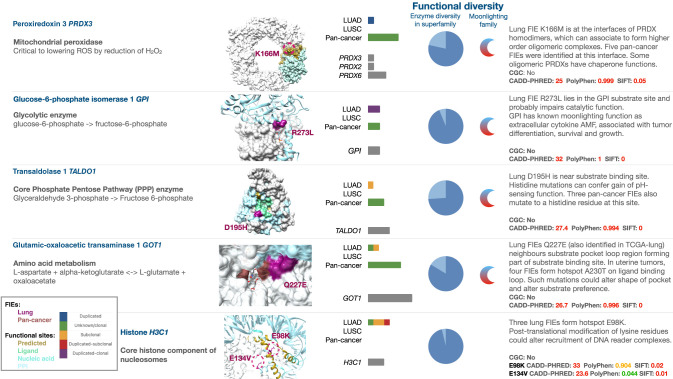
FIE genes in functionally diverse protein families are targets for discovery of potential neofunctional events late in tumor evolution. Selected examples show postduplication or subclonal lung FIEs in metabolic enzymes from functionally diverse CATH superfamilies (*PRDX3*, *GPI*, *GOT1*, and *TALDO1*) and an oncohistone (*H3C1*). Functional diversity: Enzyme diversity in superfamily indicates the number of distinct enzyme functions in the FIE's superfamily as a fraction (light blue) of the most diverse superfamily. Moonlighting family indicates when at least one gene has previously identified moonlighting functions. Bar plots indicate the distribution of FIEs identified in LUAD, LUSC, and pancancer, color-coded by the presence of gene duplication and mutation clonality as per legend. Pathogenicity predictions are shown in red (above the respective pathogenicity threshold for CADD, PolyPhen, or SIFT), in orange if they are possibly pathogenic, and in green otherwise. Oncohistones were identified by manual curation of nonmetabolic genes. Larger-sized structures are shown in Supplemental Figure 7. A complete list of 28 diverse functional families is provided in Supplemental Table 5.

From these families, seven were excluded because they predominantly contained preduplication or clonal FIEs (including those in known cancer genes *KRAS*, *BRAF*, *MAP2K1*, *STK11*, *PTPRD*, and *CDKN2A*), and a further eight were excluded when the timing was mixed or unclear (see Methods). In addition, *STK11* and *CDKN2A* are tumor-suppressor genes, in which FIEs identified known driver mutations that cause inactivation through loss of function. The remaining 13 diverse families had cancer hallmark functions, with eight metabolic, two transcription factors, a GTP binder, a histone, and a cell-cycle regulatory protein. Their FIEs represent putative novel drivers acting in later stages of lung tumor evolution. A summary of FIEs in diverse families, highlighting supporting evidence, is given in Supplemental Table 5, with case studies highlighted in Figure 4. Positive CES indicated selection of specific FIEs in these functionally diverse families, including those in genes discussed in case studies *GOT1*, *PRDX3*, and *TALDO1* (for pancancer analysis, see Supplemental Fig. 17) and, additionally, *H3C1* in TRACERx LUAD (Supplemental Fig. 18).

The potential role these FIEs might play in neofunctionalization is given in Supplemental Note 3, which presents evidence for moonlighting functions in these genes, alongside curated references to literature supporting roles in cancer. These case studies are intended to provide hypotheses for how FIEs may cause a gain or loss of function in specific proteins, complexes, or pathways, as well as the relevance this may have to lung tumor evolution.

## Discussion

This study developed a novel protocol to predict FIEs from mutations identified in tumor exome sequencing from TRACERx lung and TCGA cohort studies. Furthermore, by analyzing TRACERx tumors with copy number gains, either in specific genomic loci or following WGD, these FIEs could be timed with respect to gene duplication to provide insight into the differences between pre- and postduplication genes and functional family diversity.

A majority of FIEs were preduplication in a few known cancer genes, *TP53*, *PIK3CA*, *BRAF*, *CDKN2A*, *KRAS*, and *EGFR*, which is consistent with previous analyses of mutations in the lung (Jamal-Hanjani et al. 2017) and pancancer (Martincorena and Campbell 2015) cohorts, which identified only a select few genes with drivers in >10% of tumors in the cohort.

Gene-based *d*_N_/*d*_S_ showed strong positive selection in FIE genes owing to a high proportion of FIE genes that are known cancer genes with recurrent driver events in the tumor cohort (Fig. 2A; Supplemental Note 4). However, site-specific selection was indicated through CES analysis for FIEs more broadly, including those in genes not currently associated with cancer (Supplemental Note 5) and genes in the functionally diverse functional families (Supplemental Figs. 17–19; Supplemental Table 5) that were used to present a set of hypotheses, in the form of case studies, of potential novel late driver events in lung cancer evolution (Fig. 4; Supplemental Note 3).

Fewer FIEs were identified as postduplication than as preduplication, with more than half of these being predicted novel drivers and with each occurring in a distinct gene. This resulted in a prominent long tail in the overall FIEs per gene distribution (Fig. 3). Similar distributions have been previously observed for driver mutations, including in pancancer (Bailey et al. 2018) and prostate cancer (Armenia et al. 2018), indicating that the landscape of oncogenic drivers includes many rarely mutated genes.

Although previous studies have analyzed subclonal selection in the lung (Jamal-Hanjani et al. 2017) and other tumor types (Loeb et al. 2019), here we applied distinct approaches both to the identification of drivers (i.e., by predicting FIEs) and to their analysis with respect to gene duplication.

FunVar was designed to detect mutations with impacts on functional sites, predict rare drivers, and provide insights into the structural and functional consequences of mutations. Therefore, we made deliberate protocol design choices with specific data requirements for the detection of FIEs (i.e., functional family membership, site annotations, and mutation clustering or hotspots). Additionally, we chose a FIE score threshold that gave significant enrichment of FIE genes in known cancer genes in order to reduce the potential false-positive FIE identification rate. Although these choices reduced the number of known drivers predicted compared with other 3D detection algorithms (Supplemental Fig. 14), the benchmarking scores were comparable (Supplemental Fig. 13; Supplemental Note 1), and differences in predictions were consistent with the protocol's design (Supplemental Note 1). Both preduplication and postduplication FIEs had higher predicted pathogenicity scores than the background missense mutations.

The use of functional families permitted the confident transfer of both functional site annotations and mutations from one paralog to another in the family (Supplemental Fig. 14; Supplemental Table 11). Using this approach, we could support the validity of FIEs observed only once in the lung cohort (rare drivers) with evidence from equivalent mutations in paralogs in other cancer types, as all these FIEs were predicted to have common effects on molecular function because they were part of the same functional family. In a benchmark with known cancer driver mutations, FunVar uniquely identified drivers compared with other 3D driver detection tools, with grouping of mutations by paralogs likely contributing to detection in eight out of 19 functional families listed in Supplemental Table 11 (refer also to Supplemental Note 1; Supplemental Fig. 14).

We used a novel application of Hill–Shannon diversity metrics originally developed to measure ecological species diversity (Chao et al. 2021) to quantify the differences between the distributions of FIEs per gene and FIEs per functional family. These metrics are well suited to address difficulties in making comparisons between data sets with incomplete sampling. A tumor cohort will only provide a subset of all possible drivers for the cancer type studied, a problem analogous to that of quantifying and comparing species diversity between different habitats from samples of species abundance taken in each. The application of this Hill–Shannon method to FIEs from LUAD showed significant increases in postduplication FIE-gene and FIE-functional family diversity, relative to background mutations, compared with preduplication.

These observed increases in diversity indicate the selection for gene duplication events, which can broaden the range of possible functional adaptations available to the tumor. Somatic copy number alterations are common driver events in tumors, and copy number losses can contribute to the inactivation of tumor-suppressor genes (Davoli et al. 2013) by creating haploid regions through loss of heterozygosity (LOH) in specific alleles. Although LOH can be advantageous to a tumor, these haploid regions are susceptible to deleterious mutations in essential genes and the subsequent loss of these tumor cells under negative selection. Thus, duplication of haploid regions is thought to be one of the reasons for the high frequency (∼75% of tumors) of WGD in lung tumors (López et al. 2020; Dinh et al. 2025).

Fewer FIEs and tumors were identified as postduplication than preduplication (LUAD, 38 FIEs in 33 tumors; LUSC, 24 FIEs in 22 tumors; Fig. 1C), which given the increased postduplication FIE-gene diversity, most FIEs were only observed once in the TRACERx cohort. However, our use of functional families to identify equivalent mutations in paralogs (including other cancer types) supports the assertion of a functional effect.

Our analyses also indicated that mutation clonality is likely to play a significant role in this increased diversity, and it has recently been shown that positive selection occurs in tumors exhibiting subclonal expansion (Frankell et al. 2023). Therefore, we used clonality as an indicator of mutation timing independent of gene duplications (i.e., clonal mutations generally precede subclonal mutations) to analyze the extent to which observed increases in LUAD postduplication FIE-gene diversity could be caused by other mutational processes that were unrelated to gene duplications. Comparison of clonal and subclonal FIEs in regions with duplication to those without duplication showed increased subclonal FIE-gene diversity in duplicated regions for LUAD tumors. However, there were insufficient nonduplicated FIEs to ascertain significance at the 95% confidence level or to identify a trend for LUSC tumors, in part owing to the high proportion of tumors with WGD.

Mutational processes (unrelated to duplications) include the APOBEC cytidine deaminases that form part of the innate immune system and are associated with increased mutagenesis later in tumor evolution in many cancer types, including the lung (Roberts et al. 2013; Jamal-Hanjani et al. 2017).

Increases in mutagenesis and mutational ITH allow a greater scope for novel drivers, which will be under dynamic selective pressures from the TME and treatment regimens (Mumenthaler et al. 2015).

Candidate neofunctional FIEs were selected from highly diverse CATH superfamilies as this indicates that multiple distinct functions have arisen in these domain homologs during species evolution whereby a protein's structure changes, for example, by point mutations or the addition of extra structural motifs, “embellishments,” to a highly conserved structural core (Dessailly et al. 2010). A majority of the most diverse superfamilies have enzyme functions, and much of the functional variation arises from differences in substrate specificity, which can be altered by a single point mutation on or near the catalytic site (Das et al. 2015a). We identified four postduplication or subclonal FIEs in metabolic genes (*GPI*, *PRDX3*, *GOT1*, and *TALDO1*) in which either the protein or paralogs in the same functional family had known moonlighting functions, that is, with evidence that they could perform at least one function distinct from that of the wild type (Fig. 4; Supplemental Note 3; Supplemental Fig. 7). The presence of moonlighting functions, or secondary functions, presents a mechanism by which gene duplications can lead to neofunctionalization under the “innovation–amplification–divergence” model of neofunctionalization (Näsvall et al. 2012). In essence, a tumor may increase its adaptability to a dynamic landscape of selection pressures by exploiting an expanded repertoire of potential functional modifications. Gene duplication, allowing for amplification of a pre-existing secondary function and subsequent divergence, could be a more plausible route to new functions than de novo mutations, which usually lead to loss of function (Kuzmin et al. 2022). Identifying tumor neofunctional mutations with reference to known protein moonlighting functions is a practical search strategy used in this study, providing orthogonal evidence for neofunctional candidates to those provided by diverse CATH superfamilies.

Selected examples in which analysis of FIEs indicated putative neofunctionalization are briefly outlined below, with further examples and discussion provided in the Supplemental Materials (Fig. 4; Supplemental Note 3). We hope that these results provide helpful insights into the mechanisms by which somatic mutations can lead to neofunctionalization and interpretation of relevance in the context of tumor evolution. However, these examples were presented as a set of hypotheses and discussion points. Definitive explanations of how these FIEs affect protein function or tumor viability would require experimental studies.

In the glycolytic enzyme GPI, the potential impacts of the postduplication FIE R273L require consideration of the function of the enzyme, known associations of *GPI* mutations with cancer, and implications based on the gene's multifunctional nature. From the analysis of R273's location in the substrate site, the probable loss of H-bonds coordinating catalytic site residues caused by a mutation from arginine to leucine (i.e., loss of positively charged residue), and independent prediction of a loss of catalytic activity by MutPred2, the most likely molecular impact is loss of GPI catalytic function (Supplemental Note 3). As a core glycolytic enzyme, GPI is a crucial component of hypoxic tumor metabolism. *GPI* knockout studies in colon adenocarcinoma and mouse melanoma cell lines have shown the suppression of cell growth under hypoxic conditions (de Padua et al. 2017), and *GPI* has been implicated in conferring therapy resistance in the treatment of prostate cancer by maintaining glycolysis under hypoxia (Geng et al. 2018). However, the gene can also act as a tumor-associated extracellular cytokine, which is referred to as autocrine motility factor (*AMF*) and is associated with cell differentiation, survival, growth (Fairbank et al. 2009), and the promotion of metastases (Carlos Gallardo-Pérez et al. 2014). A discussion of this FIE in the context of substrate binding is provided in Supplemental Note 3.

In peroxiredoxins (PRDXs), the loss of a lysine residue in *PRDX3* caused by a postduplication FIE could inhibit ubiquitination and subsequent degradation that may occur under the redox conditions found within tumor cells (Nicolussi et al. 2017), thus supporting the continued removal of peroxide free radicals accumulating during tumor growth. However, the presence of five other FIEs in pancancer (Supplemental Table 4) at the same protein interface region that allows PRDX homodimers to form higher-order oligomers suggests that they might have a role in altering the stability of oligomeric PRDXs and potentially favor dimers rather than higher-order oligomers. The formation of each PRDX cysteine-cysteine disulfide bond following reduction of H_2_O_2_ requires conformational rearrangements, in which local unfolding of the alpha-helical structure in one of the dimers permits the correct atomic distance for the disulfide to be formed (Bolduc et al. 2021). All PRDX FIEs, including the one identified in LUAD, result in the loss of charged lysine, arginine, or aspartate residues, either by removing lysine or aspartate in *PRDX3;* therefore, they are likely to influence the equilibrium between dimers and higher-order oligomers formed via the affected interfaces, which in turn could influence the dynamics of helical unfolding required for catalytic function. In higher-order oligomers, some peroxiredoxins can act as chaperones (Bolduc et al. 2021). In LUAD, the identified *PRDX3* FIE creates a sulfur-containing methionine, which shares biochemical similarities with cysteine, which is able to participate in redox reactions, and has a role in cellular antioxidant defense mechanisms (Kim et al. 2014). Of note is that three out of the five pancancer FIEs in these PRDXs occurred in the paralog *PRDX6*, which is known to have moonlighting lipase and acyltransferase functions. However, FIEs are located near the peroxidatic cysteine residue forming the primary active site and not the distinct lipase/acyltransferase site. Furthermore, this distinct moonlighting site is only present in *PRDX6* and not in other paralogs (*PRDX1–5*) in the family that lack lipase/acyltransferase catalytic residues. This finding illustrates the challenging nature of neofunctional mutation prediction.

Beyond direct effects through mutations in metabolic enzymes, neofunctional mutations could occur through alterations in cell-signaling and regulatory processes, with post-translational modifications (PTMs) via phosphorylation of serine, threonine, or tyrosine residues used in intracellular signaling pathways, which are among the most widely studied examples (Hunter 1995). However, a large number of other modifications may involve other protein residues, including ubiquitination, SUMOylation, glycosylation, acetylation, and ADP-ribosylation. Therefore, one way mutations may act to change function is to create new sites of PTMs, with the most plausible example being the postduplication and subclonal FIEs in the histone H3, in which mutations to lysine could result in PTMs at a new site on this histone and alter the binding of reader complexes. Lysine mutations are important oncogenic drivers owing to their potential for multiple PTMs, including ubiquitination, SUMOylation, glycosylation, acetylation, and ADP-ribosylation (Wang et al. 2023).

The de novo computational prediction of activating kinase mutations, which includes established drivers such as *EGFR* L858R and *BRAF* V600E, remains an ongoing challenge (Jordan et al. 2019). FunVar provides a novel protocol, based on CATH functional families, to predict mutations with likely functional impact and to capture putative rare driver events occurring later in lung tumor evolution. However, definitive proof that specific FIEs drive tumor progression and the underlying mechanisms require detailed experimental characterization.

Other methods have used PPI networks or biological pathway databases to aid driver identification. Mapping mutated genes and known cancer genes to PPI networks can be used to predict drivers by inference from interacting genes and connected network components (Leiserson et al. 2015) and can incorporate additional evidence, such as known patterns of driver mutual exclusivity (Bokhari et al. 2020). Biological pathways can provide insights into the timing of driver events in a tumor cohort by mapping predicted pathogenic mutations to genes in KEGG pathways (Kanehisa et al. 2019) and estimating the order in which hallmark pathways are affected (Wang et al. 2019). These are orthogonal and complementary approaches to ours, with distinct strengths, such as not requiring protein structures, and limitations, including uncertainties arising from the use of incomplete PPI networks and a lack of site-specific predictions.

Cancer cohort studies often provide an abundance of data beyond the exonic mutations primarily used in this study, including the measurement of RNA expression, histone modification, DNA methylation, and metabolites (Chakraborty et al. 2024). Although omic approaches can be used to classify tumor subtypes and inform therapeutic decisions, the size and complexity of these data sets present analytical challenges. Current developments applying machine learning to omic data sets are an active research area that could provide novel and complementary insights to those from existing tools, such as the prediction of personalized oncogenes, tumor suppressors, or neutral genes (Sudhakar et al. 2022).

The common occurrence of WGD and chromosomal instability in lung tumors provides scope for the diversification of tumors through alterations of protein function and highlights the complex and varied avenues for adaptation to their microenvironment, immune landscape, and therapy. Through the application of a novel protocol to TRACERx genomic data, protein structure and function analysis were used to highlight increases in the diversity of predicted functional impacts later in tumor evolution. We provided a protocol for discovering novel drivers and strategies for identifying neofunctional mutations in cancer.

## Methods

### FunVar pipeline

#### Outline

We developed a Functional Variation (FunVar) protocol to identify FIEs by mapping nonsynonymous single-nucleotide variations (nsSNVs; generally referred to in text as “mutations”) to functional domain families (FunFams) defined by the CATH (v4.2) database (Sillitoe et al. 2015; Das et al. 2015b). A FunFam is a functional subclassification of homologous domains in a CATH superfamily that is predicted to have the same function and for which experimental annotation (using GO terms) has been assigned for at least one relative (Das et al. 2015b). FunVar requires each FunFam to have at least one experimentally determined 3D structure, with a single representative structure used for all members of the FunFam. All mutations and functional site annotations for a FunFam were mapped to canonical UniProt sequences and constituent superfamily domains and transferred to the representative structure via the FunFam's sequence alignment.

Pancancer mutations were obtained from 32 cancer types represented by TCGA-MC3 (about 1.4 million nonsynonymous SNVs in more than 8500 tumors) (Supplemental Table 6; Hutter and Zenklusen 2018). Experimentally confirmed functional site annotations were obtained from multiple sources: BioLip (Yang et al. 2012) ligand and nucleic acid binding sites, the Inferred Biomolecular Interaction Server (IBIS) (Shoemaker et al. 2012) PPIs, and M-CSA (Ribeiro et al. 2018) enzyme catalytic sites. Functional sites were also predicted from highly conserved positions in functional family sequence alignments, which may contain thousands of sequences from many different species. Highly conserved positions were determined using Scorecons (Valdar and Thornton 2001) as those with conservation score >0.9 in suitably diverse functional family alignments, defined as those with a diversity of position score (DOPS) > 70.

Significant clusters of pancancer mutations were identified using our previously developed MutClust algorithm (Patani et al. 2016), and the clusters were filtered to include only those occurring near (≤5 Å) a functional site. Hotspots, in which the same amino acid change at the same sequence position was observed in two or more patients with a specific cancer type, were also treated as clusters. The resulting set of mutation clusters defined residue positions that we labeled “tunable sites,” those in which mutations from different cancer types, which may be in a specific gene or in multiple paralogs containing a common functional family domain, essentially impact the same molecular function via the same functional site. A FIE in this study was a single mutation from either the TRACERx-lung, TCGA-lung, or pancancer data sets occurring within a tunable site. This type of analysis by aggregation of data is possible because of the highly coherent structure and function of relatives in the functional families in CATH (Das et al. 2021).

#### FIE score

Each FIE was scored using a simple heuristic based on mutation properties, including physicochemical shift and size change, location with respect to known and predicted functional sites, and coincidence with known disease-associated variants. The output of each score component was assigned a ranked impact value from {0,1,2}, where a score of zero indicated no impact. The component scores were grouped according to whether they were calculated at the level of protein sequence, structure, or FunFam.

The FunVar FIE score was calculated as the sum of protein sequence components (Grantham score for mutation amino acid substitution change {0,1,2}, mutation hotspot {0,1}, and known disease-associated variant {0,1}); protein structure components (on a predicted functional site {0,1}, on a known functional site {0,1}, near (≤5 Å) a predicted functional site {0,1}, near (≤5 Å) a known functional site {0,1}, or in a highly significant mutation cluster {0,1}); and a FunFam component (mutation in a mutation-enriched FunFam [MutFam] {0,1}). FIE scores are always greater than zero; by definition, FIEs must be on or near to functional sites, with scores in range [1–10].

For protein sequence components, the Grantham score was calculated using a scoring matrix (Grantham 1974) that quantified the physicochemical properties of amino acid size changes and chemical shifts. The Grantham component was scored as one for medium impact change (matrix score > 64, i.e., the median of possible Grantham scores) and as two for high impact (matrix score > 109, i.e., the upper quartile). A FIE with Grantham matrix score of 64 or less or that is polymorphic will score zero for this component, in which a polymorphic mutation is defined as one having gnomAD (Lek et al. 2016) total VAF > 10^−7^ as reported by VarMap (Stephenson et al. 2019). A hotspot mutation indicates that the same gene, residue position, and amino acid changes (e.g., *EGFR* L858R) occur more than once in the cancer type and cohort (TRACERx lung hotspots are assessed independently of any equivalent pancancer hotspots). Known disease-associated variants have a UniProt disease ID and were obtained using VarMap (Stephenson et al. 2019).

The protein structure components score for proximity (≤5 Å) to the known and predicted functional sites. FIEs that lie on a functional site were also flagged as being near the site, thus up-weighting them with a combined score of two. In addition, predicted functional sites were scored independently from known sites, resulting in a maximum overall functional site score component of four for FIEs on a known site that is also a predicted site (i.e., a highly conserved residue position in the FunFam alignment). FIEs at highly significant MutClust clusters (using significance level 0.5%; standard inclusion significance level 5%) increased the protein structure component by one.

FunFam component was scored as a one for FunFams identified as mutation-enriched FunFams using MutFam v4.0 (Ashford et al. 2019). A MutFam is a functional family previously identified to contain significantly more pancancer mutations in the domain sequence regions than nondomain regions (i.e., other protein-coding regions not part of the functional family) (Ashford et al. 2019).

The overall FIE score was used to determine a threshold score with reference to known cancer genes from the Cancer Gene Census (CGC; downloaded February 8, 2020) (Sondka et al. 2018), such that FIE genes were significantly enriched in known cancer genes (*P* < 2.2 × 10^−16^, chi-squared) (Supplemental Fig. 9; Supplemental Table 2) while still retaining FIEs in other genes not known associated with cancer, which could include novel drivers. Based on this comparison, we determined that a threshold FIE score of three provided a balance between the identification of FIEs in known cancer genes, the prediction of potential novel drivers, and filtering out those most likely to be false positives (i.e., FIEs scoring one or two) (Supplemental Fig. 9). In this study, all FIEs with a score of three or more were considered significant and were not further characterized (e.g., by ranking) according to the FIE scores. In the analysis of structures for case studies, it was helpful to include below-threshold FIEs to identify any near a functional site of interest; these below-threshold FIEs could include potential false negatives.

#### Pathogenicity predictions

Pathogenicity scores for FIEs and non-FIE missense mutations from CADD (Rentzsch et al. 2019), PolyPhen (Adzhubei et al. 2013), and SIFT (Vaser et al. 2016) were reported using VarMap (Stephenson et al. 2019).

#### Mutation timing relative to duplication

Allele-specific copy numbers and mutation copy numbers were calculated for each tumor sample, as previously described (Jamal-Hanjani et al. 2017; Gerstung et al. 2020; Frankell et al. 2023). Mutations can be timed relative to the duplication of alleles under several scenarios:

1. When a biallelic gain (MajorCN ≥ 2 and MinorCN ≥ 2) was present, a mutation copy number estimate of less than 1.5 indicated a postduplication mutation, and greater than 1.5 indicated a preduplication mutation.
2. When a monoallelic gain was present with LOH (MajorCN ≥ 2 and MinorCN = 0), a mutation copy number estimate of less than 1.5 indicated a postduplication mutation and greater than 1.5 indicated a preduplication mutation.
3. When a monoallelic gain was present without LOH (MajorCN ≥ 2 and MinorCN = 1), a mutation copy number estimate greater than 1.5 indicated a preduplication mutation; however, a mutation copy number less than 1.5 could not be timed as this could represent either a postduplication mutation on the major allele or a mutation without an associated gain on the minor allele.

In rare instances, mutations were timed postduplication in one region and preduplication in another region of the same tumor. In this case, the mutations were classified as postduplication, as once a mutation is preduplication (i.e., on all copies of an allele) wild-type allele copies cannot be generated to make the mutation appear to be postduplication. However, the reverse is possible via subclonal gain in the mutated allele copy.

### Positive selection using *d*_N_*d*_S_

Genes were analyzed for signals of positive selection using *d*_N_*d*_S_cv (Supplemental Note 4; Martincorena et al. 2017).

### CES calculations

CESs for FIEs were calculated using R package CancerEffectSizeR (v2.10.2) (Mandell et al. 2023). Effect sizes for FIEs were calculated by grouping FIEs by functional family and the mutated amino acid positions in the functional family sequence alignments. This grouping allows for CES calculations to treat equivalent mutations in paralogs within a functional family together as a “compound variant.” Detailed methods are given in Supplemental Note 5, with code and data sets in Supplemental Code 1.

### Cohorts

TRACERx421 SNVs were identified in primary tumors of 421 patients with NSCLC (LUAD: n = 235 patients, n = 239 tumors; LUSC: n = 134 patients, n = 134 tumors; other histology types: n = 46 patients, n = 46 tumors) in the TRACERx lung study (Jamal-Hanjani et al. 2017). TRACERx employs multiregion sampling of tumors, which improves detection of mutation clonality, namely, clonal mutations present in all tumor cells and subclonal mutations present in a fraction. Gene duplication and WGD events were captured and used in this study to determine the mutation timing, as outlined previously. Of the 129,315 missense mutations, 19,063 (∼14.7% of missense mutations) were mapped to PDB residues in structural representatives of CATH v4.2 FunFams. The TRACERx pipeline (Jamal-Hanjani et al. 2017) was applied retrospectively to TCGA-MC3 data to infer WGD status, clonality, and timing of mutations. TRACERx data sets are available as previously specified (Frankell et al. 2023) in the data availability section.

The pancancer data set utilized 32 cancer types available in TCGA-MC3 (Ellrott et al. 2018). We identified 1,426,100 exonic missense mutations (Supplemental Table 7), of which 224,265 were mapped to PDB residues in the structural representatives of CATH v4.2 FunFams (∼15.6%) (Supplemental Table 8). Only mutations meeting the validation thresholds (according to TCGA and TRACERx criteria) were included in the analysis.

The TCGA-lung data set is a subset of TCGA pancancer, including only LUAD and LUSC tumor types, processed using TRACERx to estimate the mutation timing, as previously described (López et al. 2020). However, only ∼30% of TCGA-lung FIEs could be timed, compared with 75% for TRACERx, with only ∼4% of TCGA-lung FIEs classified as postduplication (TRACERx 22%).

### FIE-gene diversity

FIE-gene and mutation-gene diversity scores pre- and postduplication were quantified using Hill diversity (Hill 1973) measures. To enable a comparison of Hill diversity between groups with unequal sample sizes, that is, FIEs or background mutations occurring pre- and postduplication, we employed a bootstrap subsampling (rarefication) and extrapolation method implemented in iNEXT.3D (Chao et al. 2021). This method was developed to address the equivalent problem in ecology, namely, diversity metrics will report higher diversity scores with increasing sample size, and allows for direct comparison between different samples using diversity scores and estimates of 95% CI. Hill diversity indices were calculated using either FIEs or background mutations (missense SNVs that were neither FIEs nor TRACERx drivers) per gene and compared with those found pre- or postduplication using the Shannon component (parameter *q* = 1) of Hill diversity. The Shannon diversity metric of Hill diversities was used, as it can capture both rare and common events compared with the other two metrics: species richness (Hill *q* = 0, total number of species, i.e., total gene count) or Simpson (Hill *q* = 2, weighted toward more common species, i.e., common driver genes).

Sample completeness, that is, the number of FIE genes identified compared with the theoretical maximum, can be estimated for preduplication FIEs by extrapolation of the data set size to 2*n* (where *n* is the number of genes in the number of FIEs per gene table). If the Hill diversity levels off at 2*n*, then the R/E curves can asymptotically estimate Hill diversities. However, if at 2*n* Hill diversity is increasing, then the estimates of completeness indicate a *lower* bound.

Sample coverage estimates for LUAD indicated that ∼87% of preduplication FIE genes were sampled, yielding a narrower 95% CI than for postduplication (∼50% coverage) (Supplemental Fig. 11). In addition, extrapolation of diversity scores to twice the sample size (i.e., if we theoretically identified twice the number of genes with FIEs; 2*n* sample size is recommended for iNEXT maximum when coverage is incomplete) indicated that although preduplication diversity scores start to level off (such that increasing the tumor sample size would mostly result in identifying additional FIEs in genes already sampled, such as *KRAS* and *EGFR*), those for postduplication continue to increase with no clear asymptote. This implies that the extrapolated FIE-gene diversity scores postduplication represent *lower-bound* estimates of the actual diversity.

### Identifying FIEs from diverse functional families and moonlighting paralogs

Functional diversity within a domain superfamily implies that proteins are more likely to be “tunable,” as multiple functions have arisen during evolution, while maintaining the conserved structural core of the protein domain. Functional families were considered diverse if they belonged to a CATH superfamily containing more than 10 enzyme functions, as measured by counting the number of different enzyme classification (EC) codes associated with FunFams in the superfamily. Moonlighting protein superfamilies, which contain proteins known to perform another function distinct from the wild type (such as the cytokine function of glycolytic enzyme *GPI*, or the regulation of transcription, DNA replication, and apoptosis via a distinct catalytic function of the glycolytic enzyme *GAPDH*) (Ribeiro et al. 2019; Velez Rueda et al. 2019), were obtained from MoonDB (Ribeiro et al. 2019) and ProtMiscuity (Velez Rueda et al. 2019).

FIEs were excluded from analyses if the functional family contained known cancer genes or driver mutations or protein kinases, or if the expression of the mutant allele could not be confirmed.

### Pathway analysis

Pathway enrichment analysis used sets of pre- or postduplication FIE genes tested with the g:Profiler (Raudvere et al. 2019) web tool, using the default statistical algorithm (g:SCS) and threshold (*P* < 0.05). Enrichment was identified using the GO:BP (Gene Ontology Consortium 2021), KEGG (Kanehisa et al. 2019), and Reactome pathway databases (Jassal et al. 2020).

### Protein structure analysis

Protein structures were rendered and analyzed using UCSF Chimera and ChimeraX, developed by the Resource for Biocomputing, Visualization, and Informatics at the University of California, San Francisco, with support from the National Institutes of Health R01-GM129325 and the Office of Cyber Infrastructure and Computational Biology, National Institute of Allergy and Infectious Diseases (Pettersen et al. 2021). Hydrogen bond calculations used the ChimeraX default to relax the bond constraints (distance tolerance 0.4 Å, angle tolerance 20°), as recommended for macromolecule calculations.

### Extended analysis with AlphaFold/TED protein structure models

We used the CATH domain structures from TED100, which assigns CATH domains from AlphaFold-predicted structures to domain families in CATH (Lau et al. 2024). These classifications use the most recent CATH+ version 4.3, so they were mapped to the FunVar domains from CATH v4.2, ensuring that both the UniProt sequence identifier and the segment of amino acids forming the domains matched with >80% residues in common (most of these assignments had >95% residues in common). Only domains with a single contiguous domain segment in both FunVar and TED were mapped to mitigate the introduction of artifacts from subtle differences in the CATH classification of some families between the two versions of the CATH database.

Finally, the TED100 AlphaFold-predicted structures must be of high quality to be comparable to our CATH PDB domain analysis, defined here as domains with pLDDT ≥ 90. From 6225 FunFams with more than one pancancer missense mutation, our high-quality TED100 set (“AF-TED100H”) comprised 1040 FunFams with a structure suitable for our FunVar pipeline analysis. For comparison, the number of FunFam TED100 domains with pLDDT ≥ 80 was 2197 (“AF-TED100L”). However, these lower quality models are not analyzed further here, as we do not consider them of suitable quality for comparison with the CATH PDB domains used in the main analysis. For example, low-quality structural models could be a confounding factor in the accurate determination of mutation clusters and proximity to functional sites required to identify FIEs and thus potentially novel driver events.

### Benchmarking FunVar FIEs against other 3D driver predictors

We benchmarked FunVar FIEs against existing tools for predicting cancer driver mutations using 3D protein structures. We compared the FunVar-predicted FIEs from TCGA pancancer (FIE-pancan, n = 10,828 FIEs) with driver predictions from 3dHotSpots (Gao et al. 2017), HotSpot3D (Niu et al. 2016), and HotMAPS (Tokheim et al. 2016) using the predictions provided as part of a comprehensive pancancer driver mutation and prediction study (Bailey et al. 2018) and deposited in NIH Genomic Data Commons (https://gdc.cancer.gov/about-data/publications/pancan-driver). Analysis of differences between FunVar-FIE predictions and 3D driver prediction tools (Supplemental Fig. 14; Supplemental Table 11) used the consensus of 3D scores previously generated (Bailey et al. 2018). FunVar-predicted FIEs and predictions from each of the 3D algorithms were benchmarked against independent reference sets of driver mutations from the COSMIC cancer mutation census (Tate et al. 2019) and used to define “actual positives” and likely passengers obtained from dbSNP (n = 9129 missense SNPs) (Sherry et al. 2001) and ClinVar (benign variants; n = 628 missense SNPs) (Landrum et al. 2014), as well as used to define “actual negatives.” The COSMIC cancer drivers were filtered to include only high-confidence entries that were clinically reviewed. The ClinVar data were filtered for the clinical significance “benign,” and the review status “reviewed by expert panel.” Benchmark metrics were calculated using the F1-score, accuracy, precision, and recall using R (R Core Team 2021) and using the yardstick (https://yardstick.tidymodels.org) package with resampling of the benchmark data set.

### Code availability

The software used to determine FunVar-FIE scores, diversity metrics, CESs, and benchmarking are available at GitHub (https://github.com/paulashford/funvar-tracerx) and as Supplemental Code.

## Supplemental Material

Supplement 1

Supplement 2

Supplement 3

Supplement 4
