# Supplementary material for: Gene duplication is associated with gene diversification and potential neofunctionalization in lung cancer evolution
Source: Genome Res. 2026 Mar;36(3):561–77. doi: 10.1101/gr.278663.123 (PMC12951968; doi:10.1101/gr.278663.123)
Supplement: Supplement 2 [file Supplemental_materials.pdf]

Supplemental Materials

Gene duplication is associated with gene diversification and potential neofunctionalisation in lung cancer evolution

Paul Ashford, Alexander M. Frankell, Zofia Piszka, Camilla S.M. Pang, Mahnaz Abbasian, Maise Al Bakir, Mariam Jamal-Hanjan, Nicholas McGranahan, Charles Swanton, Christine A. Orengo

Table of Contents

Table of Contents

Supplemental Note 1 Benchmarking of FunVar protocol

Outline and driver prediction tools and datasets used for comparison

Benchmarking datasets

Differences between FunVar-FIEs and cancer driver predictions

Supplemental Note 2 Extended FunFar analysis using AlphaFold protein structure models

Background

Datasets

Supplemental Note 3 Case studies of post-duplication FIEs in diverse families and potential neofunctionalisation

Histone H3

Glucose-6-phosphate isomerase

Thioredoxin-dependant peroxide reductases

Transaldolase 1

Aspartate amino acid transferase

Supplemental Note 4 Analysis of positive selection using dN/dS

Supplemental Note 5 Analysing the strength of selection of FIEs in functional families using CancerEffectSizeR

Background

Methods

CES create working sets [cesa_analysis_create_working_datasets.R]

CES analysis of functional family based compound variants sets [cesa_funfam_tcga_pancancer.R and cesa_funfam_tracerx_luad_lusc.R]

Results of CES analyses

References

Supplemental Note 1 Benchmarking of FunVar protocol

Outline and driver prediction tools and datasets used for comparison

The rise in publications depositing tumor sequencing datasets during the genomic era and the heady expansion in the volume of data this produced is well documented, leading to development of a wide range of computational tools to help mine these datasets in search of cancer drivers. While our FunVar protocol can certainly be considered a type of cancer driver prediction algorithm, our aim was not the development of a generalist driver predictor tool to compete with state-of-the-art methods. We focussed on variants that with potential to impact protein functions via functional sites and accumulated mutations from functional family paralogs predicted to share highly similar molecular functions, with particular interest in potential novel drivers post-duplication with potential for neofunctionalisation. Nonetheless, FunVar FIE benchmarks are comparable with other 3D protein structure-based tools, and reasons for differences are indicated (Supplemental Table 14).

Benchmarking datasets

In this section, we leverage datasets from existing studies that have compared multiple algorithms by predicting driver mutations across 33 tumors in TCGA and deposited the predictions. The FIEs identified in this manuscript depend on the pancancer mutation clusters identified by our FunVar protocol using the same pancancer tumor cohort as described in the study from Bailey *et al* (Bailey et al. 2018), and their study datasets are therefore an excellent resource for the purposes of our benchmarking Genomic Data Commons (https://gdc.cancer.gov/about-data/publications/pancan-driver).

While there exists no ‘gold-standard’ reference set of true driver mutations (discovery of novel drivers is ongoing and many suspected drivers have not yet been fully characterised) there are sensible strategies that can be used for benchmarking. Here, we benchmarked predictions from FunVar-FIE and each three driver predictors, HotSpot3D (Niu et al. 2016),

HotMAPS (Tokheim et al. 2016) and 3dHotSpots (Gao et al. 2017), that use protein structure against independent reference sets of driver mutations from the COSMIC cancer mutation census (Tate et al. 2019) to define “Actual Positives” and likely passengers obtained from dbSNP (Sherry et al. 2001) and benign variants from ClinVar (Landrum et al. 2014) to define “Actual Negatives”. These datasets and tools are summarised in Supplemental FIgure 12 and Methods.

Benchmark metrics were calculated, including F1-score, accuracy, precision and recall and are presented in Supplemental Figure 13. While these other methods do outperform FunVar on this classification, F1-scores are comparable (0.703 - 0.735), all tools have modest precision (0.543 - 0.581) and near perfect recall ~1. Recall scores indicate that 3D tools are not predicting SNPs or benign variants as cancer drivers.

Differences between FunVar-FIEs and cancer driver predictions

We used the consensus of 3D driver prediction scores as described in Bailey *et al* (Bailey et al. 2018) obtained from Genomic Data Commons: (https://gdc.cancer.gov/about-data/publications/pancan-driver).

These consensus score [range 0 - 4] identified commonly mutated residues from four 3D methods, HotSpot3D (Niu et al. 2016), HotMAPS (Tokheim et al. 2016) and 3dHotSpots (Gao et al. 2017) and eDriver (Porta-Pardo and Godzik 2014).

There were two reasons why mutations were predicted to be cancer drivers by other 3D methods but not as FIEs by FunVar: firstly, the stricter data requirements for FunVar, as mutations must have been assigned to a functional family with functional site annotations, in addition to the requirement for protein structures; secondly, the protocol was designed and implemented to capture mutations affecting functional sites through the identification of significant mutation clustering. Using the COSMIC Cancer Mutation Census as a reference for known drivers, we identified distinct protein residues commonly identified by 3D methods

and FunVar, uniquely identified only by other methods and those unique to FunVar (Supplemental Figure 14 and Supplemental Table 11). Where mutations were classed as drivers only by other methods and not as FIEs, this was because mutations could not be assigned to a functional family or domain structure (30% of cases), were assigned to families with limited or no functional site annotations (32% of cases), and where no significant mutation clusters were identified (35% cases) (Supplemental Figure 14). The majority of mutated protein sites where no significant cluster was identified (i.e., MutClust clusters were not significant at level 5%) were from mutations in tumor suppressor genes, predominantly *TP53*, *PTEN* and *VHL* (Supplemental Figure 14). FIEs were uniquely identified in 19 functional families (Supplemental Table 11), with grouping of paralogs and accumulation of mutations likely contributing to FIEs where other 3D tools do not predict drivers in 8 families.

Supplemental Note 2 Extended FunFar analysis using AlphaFold protein structure models

Background

The release of AlphaFold (Jumper et al. 2021) had major implications for protein structural biology, with the latest release holding over 200 million entries (Varadi et al. 2024), expanding coverage of the structural universe in a short space of time, with clear implications for structure-function relationships, drug-discovery, treatment of diseases and metagenomics.

While the AlphaFold releases were too late in the development of our FunVar pipeline to be included from the outset, we address here the findings from an extended analysis using high quality AlphaFold models for CATH functional family domains that did not have an available PDB structure in the original analysis.

An important caveat to this is that our FunVar protocol identifies FIEs occurring in clusters (or hotspots) near to functional sites. These sites can either be ‘known’, i.e. previously identified by analysis of PDB protein structures (here: catalytic sites, ligand and nucleic acid binding sites, and protein interface residues). However, for AlphaFold models of CATH FunFam domains that had no available PDB domain structure will not have any annotations of these ‘known’ functional sites with which to define FIEs. This lack of functional sites is simply due to the fact that when a functional family has no PDB domain structure available as a representative, this is because none of the family members (i.e., homologues) had a structure to choose from.

In essence, the following analysis has a simple symmetry: FIEs identified in functional families using predicted protein structures from AlphaFold are identified with reference to predicted functional sites (Scorecons90, highly conserved positions in the family sequence alignment, see Methods).

Datasets

Missense SNVs from TCGA pancancer were grouped into sets of paralogs using CATH functional families and accumulated to a single functional family representative protein sequence using the same protocol used for PDB domain analysis (see Methods). AlphaFold (Jumper et al. 2021) structures for these sequence domains were assigned using The Encyclopedia of Domains (TED) (Lau et al. 2024).

We used CATH domain structures from TED100, which assigns CATH domains from AlphaFold models to protein sequence clusters with 100% sequence identity (as described in (Lau et al. 2024)). These classifications use the most recent CATH+ version 4.3, so these were mapped to the FunVar domains from CATH v4.2 by ensuring that both the UniProt sequence identifier and the segment of amino acids forming the domains matched with >80% residues in common (most of these assignments had >95% residues in common). Only domains having a single contiguous domain segment in FunVar both and TED were mapped, to mitigate the introduction of artefacts from subtle differences in the CATH classification of some families between the two CATH-db versions.

Finally, the TED100 AlphaFold model must have high quality to be comparable with our CATH PDB domain analysis, defined here as domains with pLDDT>=90. From 6,225 FunFams having >1 pancancer missense mutation, our high quality TED100 set (“AF-TED100H”) comprised 1,040 FunFams with a structure suitable for our FunVar pipeline analysis. For comparison, the number of FunFam TED100 domains with pLDDT >=80 is 2,197 (“AF-TED100L”). However, these lower quality models are not analysed further here, as we do not consider them of suitable quality to compare with CATH PDB domains used in the main analysis. For example, low quality structure models would be a confounding factor in the accurate determination of mutation clusters and proximity to functional sites required to identify FIEs and thus potentially novel driver events.

FunVar scoring was run on mutations mapped to AlphaFold/TED for pancancer and TRACERx. TRACERx FIEs from these modeled domains are included in Supplemental Table 10. In addition, the Hill-Shannon diversity calculations were extended with additional FIEs as discussed in the main text.

Supplemental Note 3 Case studies of post-duplication FIEs in diverse families and potential neofunctionalisation

These examples present discussions on some of the post-duplication and subclonal FIEs identified in TRACERx lung tumors and hypotheses on how these could alter protein function (**Figure 4, Supplemental Table 5**).

Histone H3

In Histone H3, a core part of the nucleosomes, three subclonal FIEs (one of which is confirmed post-duplication) form an E98K hotspot. This mutation of glutamic acid to lysine is potentially neofunctional as it could lead to abnormal recruitment of reader complexes following post-translational modification (PTM) of the lysine, as suggested by Nacev *et al* (Nacev et al. 2019). In addition, their study found E98K was the most frequently mutated residue in Histone H3 where over half of mutations were to a lysine residue at or near known post-translational modification sites, where modifications such as mono-, di- or tri-methylation or acetylation can alter recognition by regulatory or transcriptional proteins (Nacev et al. 2019).

Glucose-6-phosphate isomerase

The cytoplasmic enzyme glucose-6-phosphate isomerase (*GPI*) catalyses the conversion of glucose-6-phosphate to fructose-6-phosphate, forming the second step in the glycolytic pathway forming two molecules of pyruvate from each one of glucose.

In addition, *GPI* can function as a tumor associated cytokine following release into the extracellular matrix, where it is referred to as autocrine motility factor (AMF). AMF is considered to be a moonlighting function of *GPI*, with respect to its primary glycolytic role.

We identified an expressed, post-duplication FIE (R273L) in a LUAD tumor which results in removal of an arginine residue forming part of the glucose-6-phosphate binding site (**Figure 4**), which partially overlaps the cytokine receptor (AMFR) binding site (Tanaka et al. 2002). A FIE at the same position in this site (R273C) was also identified in a colorectal tumor in the TCGA pan-cancer dataset.

Due to proximity to the *GPI* catalytic site residues, this LUAD FIE would be likely to impair enzymatic function, a prediction supported by the association of mutations at this residue with chronic haemolytic anaemia (Xu and Beutler 1994), a disease which can be caused by *GPI* dysfunction.

We analysed the extent to which FIE R273L might impact H-bonds between substrate binding site residues and the *GPI* glycolytic substrate, glucose-6-phosphate, compared with those of an inhibitor of the AMF moonlighting function of *GPI*, erythose-4-phosphate. With the *GPI* substrate, glucose-6-phosphate, we identified a H-bond to R273 (**Supplementary Figure 5**). However, analysing the AMF inhibitor binding site we found no predicted H-bond between erythose-4-phosphate and R273 (**Supplementary Figure 5**). The observed differences in active site H-bonding to residue R273 between these two substrates was likely to be due to the different sizes of the two molecules. The AMF-inhibitor erythose-4-phosphate was smaller than glucose-6-phosphate, resulting in fewer H-bonds to active site residues in the deeper parts of the pocket where R272 was situated. It is therefore possible that FIE R273L has more impact on the glycolytic function of GPI compared with the moonlighting AMF function. Independent prediction of mutation R273L impact with MutPred2 (Pejaver et al. 2020) predicted pathogenicity (MutPred2 score=0.937) with top three functional impacts: (1) altered transmembrane protein, (2) altered ordered interface and (3) loss of catalytic activity (all with $P \leq 1.6x10^{-3}$).

Given that FIE R273L occurred post-duplication, it is possible that both glycolysis and tumor hypoxic metabolism are supported via functional *GPI* coded on the non-mutant allele. Our

predictions of the impact of mutation R273L using protein structure indicate a loss-of catalytic function, and mutations at this site are associated with the germline disease chronic hemolytic anaemia (Xu and Beutler 1994) (a condition that can arise from a *GPI* deficiency), so it is most likely that the mutant allele does not support *GPI* function. However, R273L mutants could still support the tumor-associated cytokine AMF function. While there is general literature consensus that the AMFR and *GPI* sites at least partly overlap (Tanaka et al. 2002), our analysis of H-bonding in the substrate binding site indicate that although there is a prominent role for R273 in both active site coordination and direct H-bonding to the glycolytic substrate glucose-6-phosphate, this is not the case for erythrose-4-phosphate, an inhibitor of AMF function.

Thioredoxin-dependant peroxide reductases

The peroxiredoxins, including human *PRDX1-6*, are a class of thioredoxin-dependent peroxide reductases catalysing the breakdown of hydrogen peroxide (H_2O_2) and other reactive oxygen species that are generated during oxidative metabolism. The peroxiredoxins are highly conserved across eukaryotes, prokaryotes and archaea (Bolduc et al. 2021).

We identified a post-duplication FIE K166M in the mitochondrial *PRDX3*. This FIE was located near a highly conserved site within the functional family and in the same region as 5 other FIEs identified in other cancer types from the pan-cancer dataset in *PRDX3* and paralogs *PRDX6* and PRDX2 (Supplementary Figure 6 and Supplementary Table 4). PRDX3 is a typical 2-Cysteine peroxiredoxin that functions via a pair of catalytic cysteine residues, one from each subunit of an obligate homodimer complex. The peroxidatic cysteine from one subunit reduces H_2O_2 and the oxidised cysteine subsequently forms a disulphide bond with the resolving cysteine from the other subunit (Bolduc et al. 2021). The homodimeric PRDXs associate into higher order structures forming decamers or dodecamers and it is at the interfaces between these higher order complexes that the LUAD FIE and all 5 pan-cancer FIEs are found.

Transaldolase 1

The transaldolase *TALDO1* has been identified with a 5 gene 'metabolic cluster' recently identified as a predictor of poor survival in triple-negative breast cancers (Aslan et al. 2021) and germline variants with an increased risk of head and neck squamous cell carcinomas (Basta et al. 2008). *TALDO1* plays a role in the pentose phosphate pathway (PPP), providing supplies of both nucleotides for growth and NADPH as a precursor to glutathione-dependent oxidative stress responses. What was most striking about the D195H FIE, identified in LUAD, and the three other *TALDO1* FIEs identified in the pancancer dataset, was that all created histidine residues at a site neighbouring the substrate binding pocket. Histidine residues are sensitive to changes in cellular pH due to histidine's imidazole ring, which allows for gain or loss of a proton at around cellular pH. This pH-dependent histidine protonation is exploited as part of the catalytic mechanisms of many enzymes, via shuttling of protons from a donor amino acid to an acceptor (Li and Hong 2011) and additionally in cancer cells can act as a buffer for tumor cells due to the increased proton flux generated during aerobic glycolysis (the 'Warburg effect') which results in a lower extracellular pH and higher intracellular pH compared to non-cancer cells (Cardone et al. 2005). In addition to pH-dependent buffer effects, these four FIEs would be likely to alter H-bonding interactions at the substrate binding site, as D195H changes negatively charged aspartate to histidine and R239H changes the positively charged arginine to a pH-dependent histidine. A gain of pH-sensing function has been shown for histidine mutations in *TP53* and *EGFR* (White et al. 2017), resulting in tumor cells becoming sensitive to increased intracellular pH via the deprotonation of mutant histidine residues, resulting in reduced p53-DNA binding affinity or increased stability of an active conformation of *EGFR* kinase domain (White et al. 2017). It has also been shown via mutagenesis that it is possible to change the function of transaldolase to that of fructose-6-phosphate aldolase with a single mutation at the substrate site (Schneider et al. 2008) (though not residue D195). Such a neofunctional mutation would permit generation of glycolytic intermediate D-glyceraldehyde-phosphate directly from

fructose-6-phosphate. The association of *TALDO1* mutations with cancer risk and survival outcomes and the neofunctional potential indicated by mutagenesis studies indicates that mutations in *TALDO1* are worthy of further investigation.

Aspartate amino acid transferase

The aspartate amino acid transferase *GOT1* catalyses the reversible conversion of L-aspartate and alpha-ketoglutarate with L-glutamate and oxaloacetate. These enzymes are part of a family with highly conserved active site sequences and structures that catalyse a variety of amino acids and ketoacids in a pyridoxal phosphate (PLP)-dependent manner and are crucial components of the Krebs and Urea cycles. A subclonal FIE in LUAD (*GOT1* Q227E), also identified in the TCGA-lung cohort, is near to a ligand binding site on a loop forming the substrate pocket, and neighbours a substrate binding residue (Y226). Such mutations could influence the shape of the ligand pocket and thus affect substrate preference. A further hotspot (A230T) was identified in 4 uterine tumors affecting the same ligand binding loop.

Supplemental Note 4 Analysis of positive selection using dN/dS

We assessed dN/dS using R package $d_N d_S$cv (Martincorena et al. 2017) , which accounts for differences in mutation rates between and within genes and the trinucleotide context of mutations, using package release dndscv.0.1.0 from https://github.com/im3sanger/dndscv. Variant Call Files (VCFs) suitable for $d_N d_S$cv were generated from the TRACERx GRCh37/hg19 mutation table with output columns: sample_id (using the tumour_id), chromosome number, reference allele and variant allele. FIE-genes comprised 109 distinct genes from all 355 TRACERx FIEs. Global dN/dS for all mutation types in the TRACERx VCF was run using a targeted gene list option with 109 FIE genes (Figure 2a). Note that while FIE genes use the latest HUGO nomenclature, 7 gene names (*H2BC4, H2BC9, H3C11, H3C12, H2BC26, POGLUT2, TNS2)* were modified in the input to match previous names in $d_N d_S$cv RefCDS.

We then ran $d_N d_S$ for mutations mapped to functional families that had functional site annotation and a 3D structure (i.e., missense SNVs within the scope for prediction as FIEs, plus synonymous SNVs in the same scope) using FIE-genes that were known CGC cancer genes (n=25 genes) or not (n=84 genes).

**TRACERx mutations (FunFam mapped)**	**$d_N d_S$ maximum likelihood estimation (95% CI) for missense SNVs**
All	1.0456 (1.0170, 1.0751)
FIE-genes CGC	1.8703 (1.6068, 2.1769)
FIE-genes not CGC	1.0812 (0.8893, 1.3145)

FIEs in known cancer genes provide the dominant signal of positive selection. Given that typically a non cancer gene will have only a single FIE predicted, dN/dS calculations will tend

to be dominated by the larger number of missense variants in the gene that are not FIEs. In addition, it is more likely that genes showing strong positive selection for missense variants would already be included in the CGC.

Supplemental Note 5 Analysing the strength of selection of FIEs in functional families using CancerEffectSizeR

Background

CancerEffectSizeR (CES) is an R package that can estimate the strength of selection for specific mutations in a tumor cohort by rigorously accounting for variations in mutation rates in the genome, for different tissue types, and for tumor samples based on mutational signature analyses (Mandell et al. 2023).

CES (v2.10.2) analysis was applied to FIEs in the context of their functional families through the use of the ‘compound variant set’ functionality provided by the package. Compound variant sets allow grouping of arbitrary sets of mutations, which are then treated together as single variants for calculation of cancer effect size. We defined a compound variant as any mutations found in TCGA pan-cancer or TRACERx-lung cohorts that occurred at the same protein amino acid position using the functional family sequence alignments to include equivalently mutated residues in paralogs in the compound variant sets (TCGA pan-cancer and TRACERx-lung were each analysed separately).

Methods

Code and data from this analysis is provided under ‘Software Availability’ as supplemental code files (Supplemental Code 1**)**. We applied CES analysis to TRACERx-lung and TCGA-pancancer data according to the authors’ vignette:

https://townsend-lab-yale.github.io/cancereffectsizeR/articles/cancereffectsizeR.html

CES create working sets [cesa_analysis_create_working_datasets.R]

For each of the cohorts TCGA pan-cancer, TRACERx LUAD, and TRACERx LUSC we created a CES ‘working set’ (one per cohort) by converting tab-separated mutation tables to mutation annotation files (MAF) representing whole-exome coverage of SNVs, loading each MAF into a CES data structure and referencing against human genome build hg19 (provided by CES as “ces.refset.hg19” (v1.1.3)).

Site and tumor sample specific mutation rates were calculated with a mutational signature analysis using MutationalPatterns (Blokzijl et al. 2018), which in the cases of TRACER LUAD and LUSC, excluded signatures not usually found in each of the lung histology types. Mutation rates per gene were calculated with $d_N d_S$cv (Martincorena et al. 2017).

CES analysis of functional family based compound variants sets [cesa_funfam_tcga_pancancer.R and cesa_funfam_tracerx_luad_lusc.R]

The CES analysis of working sets proceeds by grouping each FIE by both functional family and residue position in the family representative protein domain. This approach groups mutations in paralogs at equivalent alignment positions together as compound variants and allows CES to calculate cancer effects across the functional family paralogs used by FunVaar. These two scripts handle the creation of data tables required for the plots of Supplemental Figures 15, 16, 17, 18 & 19.

Results of CES analyses

We selected five subsets of the FIE TRACERx and TCGA pan-cancer datasets to provide evidence that FIEs identified by our FunVar protocol have non-zero cancer effect sizes, which with reference to polymorphic mutations from dbSNP annotated as benign/non-disease-causing indicate positive selection in variants annotated as FIEs. Three figures show cancer effect sizes from analyses of TCGA pan-cancer data (Supplemental Figure 15, Supplemental Figure 16, and Supplemental Figure 17). A further two figures show cancer

effect sizes for the functionally diverse functional families given in Supplemental Table 5 for TRACERx LUAD (Supplemental Figure 18) and TRACERx LUSC (Supplemental Figure 19).

Supplemental Figure 15 shows cancer effect sizes for pan-cancer mutations grouped by functional family and mutated residue position for FIEs uniquely identified by FunVar in the benchmarking tests (Supplemental Table 11 and Supplemental Note 1). Supplemental Figure 17 shows pan-cancer cancer effect size for the top functionally diverse FIE-containing functional families in Supplemental Table 5. In comparison, Supplemental Figure 16 shows cancer effect sizes for dbSNP benign variants found in the TCGA pan-cancer dataset (and also mapped to FunFams and protein structures, as per the FunVar protocol). The unitless cancer effect sizes for dbSNP variants are predominantly low, with 9/25 functional families having CES score of ~1 or lower, with a further 13/25 families having scores 10 or lower (as measured by the lower of the 95%CI for the highest effect variant in the functional family). Low cancer effect scores are expected for this dbSNP polymorphism dataset. In contrast, pan-cancer FIEs shown in Supplemental Figure 15 all have positive CES scores (0/18 functional families with best CES ~ 1 or lower; 3/18 families with best CES <10 and 5/18 families with CES scores ~100 or higher). For the functionally diverse families in Supplemental Table 5, CES scores per FunFam (Supplemental Figure 17) (0/24 functional families with best CES ~ 1 or lower; 8/24 families with best CES <10 and ~10 families with CES scores ~100).

Cancer effect sizes are greater overall for TRACERx LUAD (Supplemental Figure 18) and LUSC (Supplemental Figure 19) than TCGA pan-cancer, likely due in part to the TRACERx CES analysis using both lung-tissue specific covariates for the gene-based $d_N d_S$cv analysis, and the exclusion of non-lung mutational signatures from the mutational processes analysis; together these provide better discernment of mutation selection effects from background mutational process.

References

Aslan M, Hsu E-C, Garcia-Marques FJ, Bermudez A, Liu S, Shen M, West M, Zhang CA, Rice MA, Brooks JD, et al. 2021. Oncogene-mediated metabolic gene signature predicts breast cancer outcome. *Npj Breast Cancer* **7**: 1–13.

Bailey MH, Tokheim C, Porta-Pardo E, Sengupta S, Bertrand D, Weerasinghe A, Colaprico A, Wendl MC, Kim J, Reardon B, et al. 2018. Comprehensive Characterization of Cancer Driver Genes and Mutations. *Cell* **173**: 371-385.e18.

Basta PV, Bensen JT, Tse C-K, Perou CM, Sullivan PF, Olshan AF. 2008. Genetic variation in Transaldolase 1 and risk of squamous cell carcinoma of the head and neck. *Cancer Detect Prev* **32**: 200–208.

Blokzijl F, Janssen R, Van Boxtel R, Cuppen E. 2018. MutationalPatterns: comprehensive genome-wide analysis of mutational processes. *Genome Med* **10**: 33.

Bolduc J, Koruza K, Luo T, Malo Pueyo J, Vo TN, Ezeriņa D, Messens J. 2021. Peroxiredoxins wear many hats: Factors that fashion their peroxide sensing personalities. *Redox Biol* **42**: 101959.

Cardone RA, Casavola V, Reshkin SJ. 2005. The role of disturbed pH dynamics and the Na+/H+ exchanger in metastasis. *Nat Rev Cancer* **5**: 786–795.

Gao J, Chang MT, Johnsen HC, Gao SP, Sylvester BE, Sumer SO, Zhang H, Solit DB, Taylor BS, Schultz N, et al. 2017. 3D clusters of somatic mutations in cancer reveal numerous rare mutations as functional targets. *Genome Med* **9**: 4.

Jumper J, Evans R, Pritzel A, Green T, Figurnov M, Ronneberger O, Tunyasuvunakool K, Bates R, Žídek A, Potapenko A, et al. 2021. Highly accurate protein structure prediction with AlphaFold. *Nature* **596**: 583–589.

Landrum MJ, Lee JM, Riley GR, Jang W, Rubinstein WS, Church DM, Maglott DR. 2014. ClinVar: public archive of relationships among sequence variation and human phenotype. *Nucleic Acids Res* **42**: D980-985.

Lau AM, Bordin N, Kandathil SM, Sillitoe I, Waman VP, Wells J, Orengo CA, Jones DT. 2024. Exploring structural diversity across the protein universe with The Encyclopedia of Domains. *Science* **386**: eadq4946.

Li S, Hong M. 2011. Protonation, Tautomerization, and Rotameric Structure of Histidine: A Comprehensive Study by Magic-Angle-Spinning Solid-State NMR. *J Am Chem Soc* **133**: 1534–1544.

Mandell JD, Cannataro VL, Townsend JP. 2023. Estimation of Neutral Mutation Rates and Quantification of Somatic Variant Selection Using cancereffectsizeR. *Cancer Res* **83**: 500–505.

Martincorena I, Raine KM, Gerstung M, Dawson KJ, Haase K, Van Loo P, Davies H, Stratton MR, Campbell PJ. 2017. Universal Patterns of Selection in Cancer and Somatic Tissues. *Cell* **171**: 1029-1041.e21.

Nacev BA, Feng L, Bagert JD, Lemiesz AE, Gao J, Soshnev AA, Kundra R, Schultz N, Muir TW, Allis CD. 2019. The expanding landscape of ‘oncohistone’ mutations in human

cancers. *Nature* **567**: 473–478.

Niu B, Scott AD, Sengupta S, Bailey MH, Batra P, Ning J, Wyczalkowski MA, Liang W-W, Zhang Q, McLellan MD, et al. 2016. Protein-structure-guided discovery of functional mutations across 19 cancer types. *Nat Genet* **48**: 827–837.

Pejaver V, Urresti J, Lugo-Martinez J, Pagel KA, Lin GN, Nam H-J, Mort M, Cooper DN, Sebat J, Iakoucheva LM, et al. 2020. Inferring the molecular and phenotypic impact of amino acid variants with MutPred2. *Nat Commun* **11**: 5918.

Porta-Pardo E, Godzik A. 2014. e-Driver: a novel method to identify protein regions driving cancer. *Bioinformatics* **30**: 3109–3114.

Schneider S, Sandalova T, Schneider G, Sprenger GA, Samland AK. 2008. Replacement of a Phenylalanine by a Tyrosine in the Active Site Confers Fructose-6-phosphate Aldolase Activity to the Transaldolase of Escherichia coli and Human Origin. *J Biol Chem* **283**: 30064–30072.

Sherry ST, Ward M-H, Kholodov M, Baker J, Phan L, Smigielski EM, Sirotkin K. 2001. dbSNP: the NCBI database of genetic variation. *Nucleic Acids Res* **29**: 308–311.

Tanaka N, Haga A, Uemura H, Akiyama H, Funasaka T, Nagase H, Raz A, Nakamura KT. 2002. Inhibition Mechanism of Cytokine Activity of Human Autocrine Motility Factor Examined by Crystal Structure Analyses and Site-directed Mutagenesis Studies. *J Mol Biol* **318**: 985–997.

Tate JG, Bamford S, Jubb HC, Sondka Z, Beare DM, Bindal N, Boutselakis H, Cole CG, Creatore C, Dawson E, et al. 2019. COSMIC: the Catalogue Of Somatic Mutations In Cancer. *Nucleic Acids Res* **47**: D941–D947.

Tokheim C, Bhattacharya R, Niknafs N, Gygax DM, Kim R, Ryan M, Masica DL, Karchin R. 2016. Exome-scale discovery of hotspot mutation regions in human cancer using 3D protein structure. *Cancer Res* **76**: 3719–3731.

Varadi M, Bertoni D, Magana P, Paramval U, Pidruchna I, Radhakrishnan M, Tsenkov M, Nair S, Mirdita M, Yeo J, et al. 2024. AlphaFold Protein Structure Database in 2024: providing structure coverage for over 214 million protein sequences. *Nucleic Acids Res* **52**: D368–D375.

White KA, Ruiz DG, Szpiech ZA, Strauli NB, Hernandez RD, Jacobson MP, Barber DL. 2017. Cancer-associated arginine-to-histidine mutations confer a gain in pH sensing to mutant proteins. *Sci Signal* **10**: eaam9931.

Xu W, Beutler E. 1994. The characterization of gene mutations for human glucose phosphate isomerase deficiency associated with chronic hemolytic anemia. *J Clin Invest* **94**: 2326–2329.
