# Supplementary material for: Gene duplication is associated with gene diversification and potential neofunctionalization in lung cancer evolution
Source: Genome Res. 2026 Mar;36(3):561–77. doi: 10.1101/gr.278663.123 (PMC12951968; doi:10.1101/gr.278663.123)
Supplement: Supplement 3 [file Supplemental_Figures.pdf]

Supplemental Figures

Gene duplication is associated with gene diversification and potential neofunctionalisation in lung cancer evolution

Paul Ashford, Alexander M. Frankell, Zofia Piszka, Camilla S.M. Pang, Mahnaz Abbasian, Maise Al Bakir, Mariam Jamal-Hanjan, Nicholas McGranahan, Charles Swanton, Christine A. Orengo

Table of Contents

Supplemental Figures i

Table of Contents ii

Supplemental Figure 1 Germline mutability of FIE-genes of other genes in NSCLC 1

Supplemental Figure 2 Long tail distributions of FIE counts per gene 2

Supplemental Figure 3 FIE-functional family diversity in LUSC 3

Supplemental Figure 4 FIE-gene diversity for clonal and subclonal events in regions with and without gene duplications 4

Supplemental Figure 5 Comparison of H-bonds in region of substrate pocket, substrate and AMF-inhibitor near location of LUAD FIE at Arg272. 5

Supplemental Figure 6 Location of FIEs at oligomeric interfaces of PRDXs 6

Supplemental Figure 7 Larger images for selected example structures from Figure 4 7

Supplemental Figure 8 Most diverse CATH superfamilies by number of functional families 8

Supplemental Figure 9 Number of pan-cancer FIEs identified by FunVar score threshold 9

Supplemental Figure 10 FIEs identified pre- and post-duplication for LUAD/LUSC in TRACERx and TCGA-lung cohorts 10

Supplemental Figure 11 Sample coverage estimates for LUAD FIE-gene diversity analysis 11

Supplemental Figure 12 Datasets used for FunVar-FIE benchmarking 12

Supplemental Figure 13 FunVar-FIE benchmarking scores compared to cancer driver predictors using 3D protein structures 13

Supplemental Figure 14 Summary of predictions from FunVar-FIE (pan-cancer) and other 3D tools on mutations in reference set comprising known cancer driver mutations 14

Supplemental Figure 15 Cancer effect size for pan-cancer functional families with FunVar uniquely predicted drivers in benchmarking 15

Supplemental Figure 16 Cancer effect size for dbSNP benign variants in TCGA pan-cancer, grouped by functional family 16

Supplemental Figure 17 Cancer effect size for top functionally diverse FIE-containing functional families 17

Supplemental Figure 18 Cancer effect size for top functionally diverse families in TRACERx LUAD 18

Supplemental Figure 19 Cancer effect size for top functionally diverse families in TRACERx LUSC 19

Supplemental Figure 1 Germline mutability of FIE-genes of other genes in NSCLC

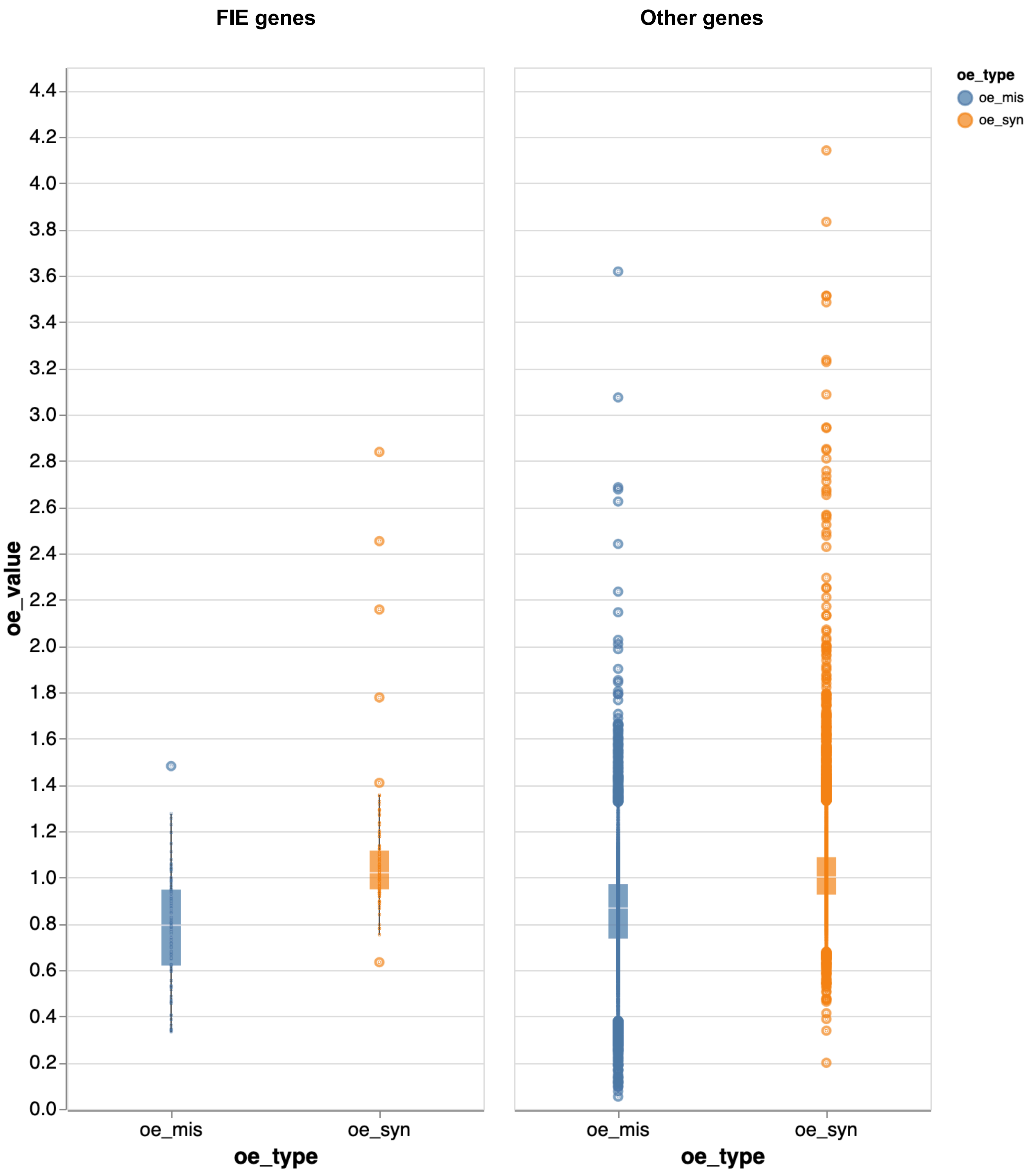

Observed/expected (o/e) mutation values per gene, obtained from gnomAD, indicate depletion for germline missense variants (*blue*) in all genes compared to synonymous (*yellow*).
FIE-genes have significantly lower o/e values for missense mutations than other-genes (*P*=0.002, Welch's *t*-test) indicating negative selection for missense variants in these genes in the germline.
Numerical annotations give Mean (±SD).

Supplemental Figure 2 Long tail distributions of FIE counts per gene

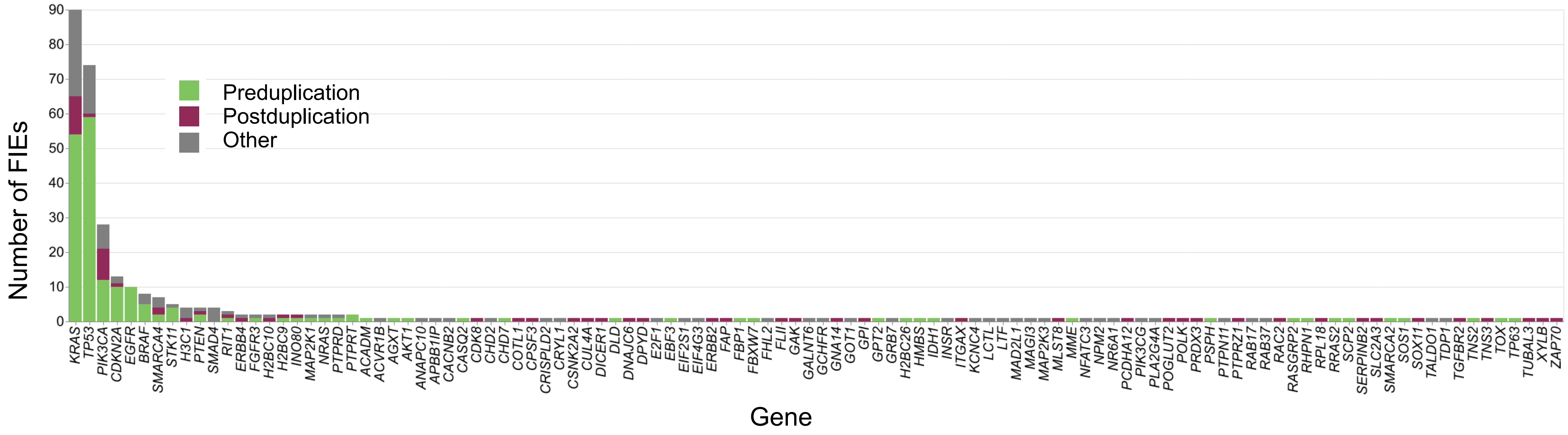

A majority of FIEs identified in the cohort of LUAD and LUSC tumors were in a few predominant driver genes, followed by a pronounced long-tail comprising many genes with only a single FIE identified in each.
(This is a larger format version of Figure 3D)

Supplemental Figure 3 FIE-functional family diversity in LUSC

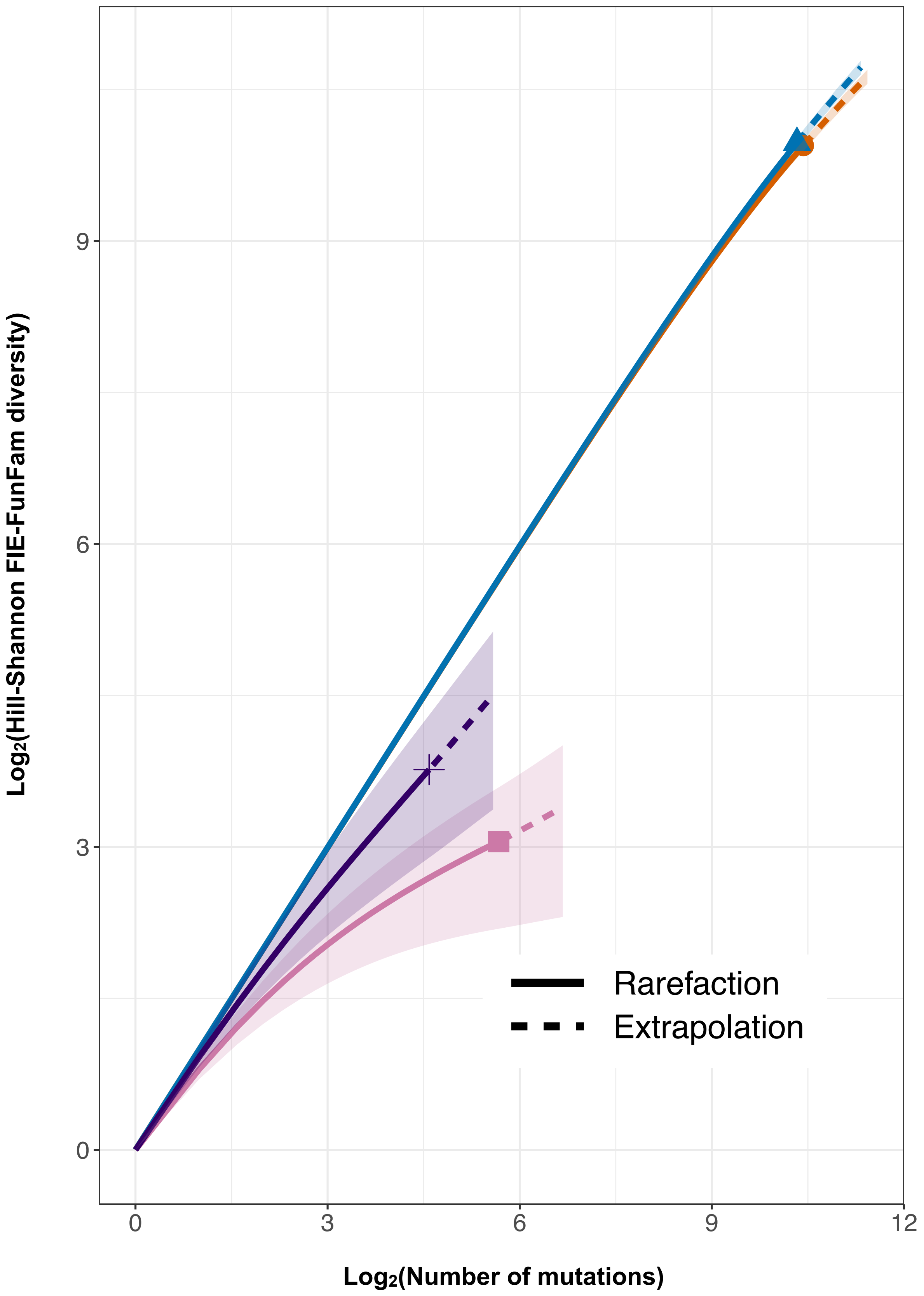

Supplemental Figure 4 FIE-gene diversity for clonal and subclonal events in regions with and without gene duplications

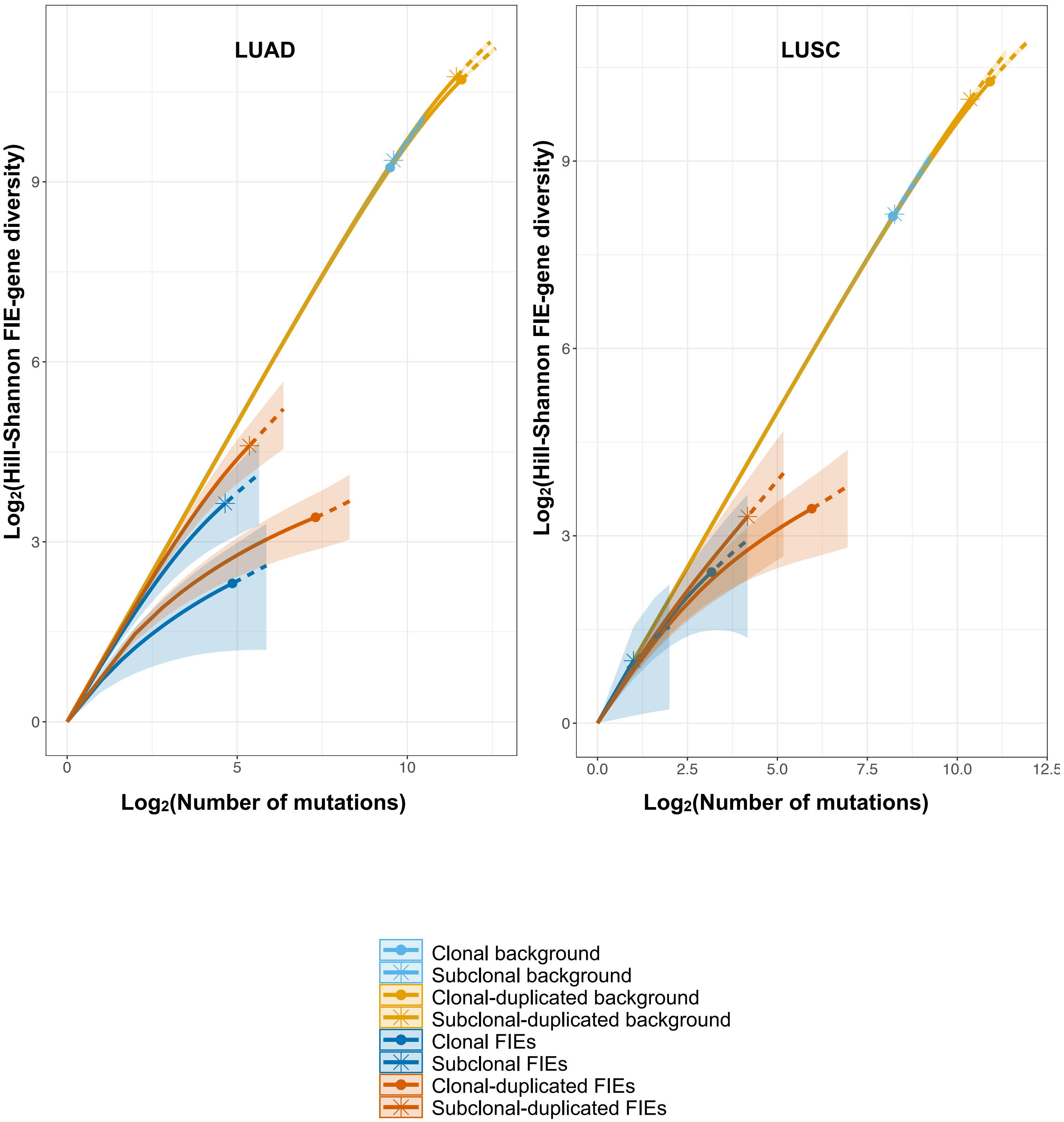

LUAD subclonal FIEs-gene diversity is higher than clonal FIEs-gene diversity in duplicated and non-duplicated regions. Duplication may act to increase FIE-gene diversity, especially for subclonal FIEs, but this could not be confirmed significant at 95%CI. The small number of FIEs in non-duplicated regions leads to correspondingly wide confidence intervals for diversity estimates in these cases (*blue*). Insufficient numbers of FIEs were identified in duplicated and non-duplicated regions in LUSC.

Supplemental Figure 5 Comparison of H-bonds in region of substrate pocket, substrate and AMF-inhibitor near location of LUAD FIE at Arg272.

A

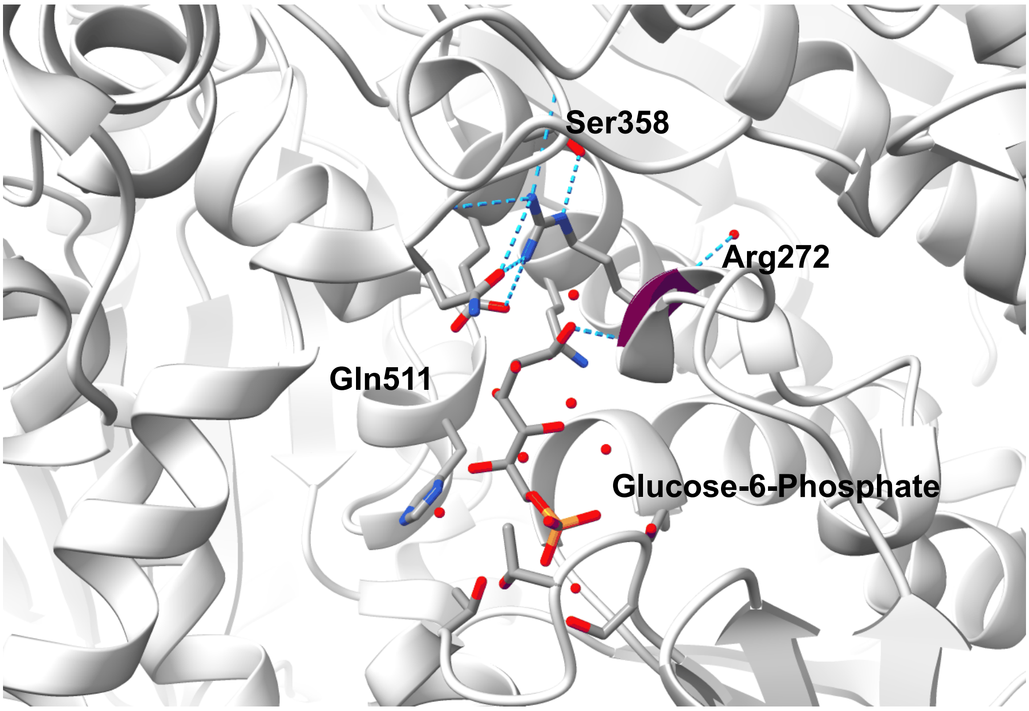

B

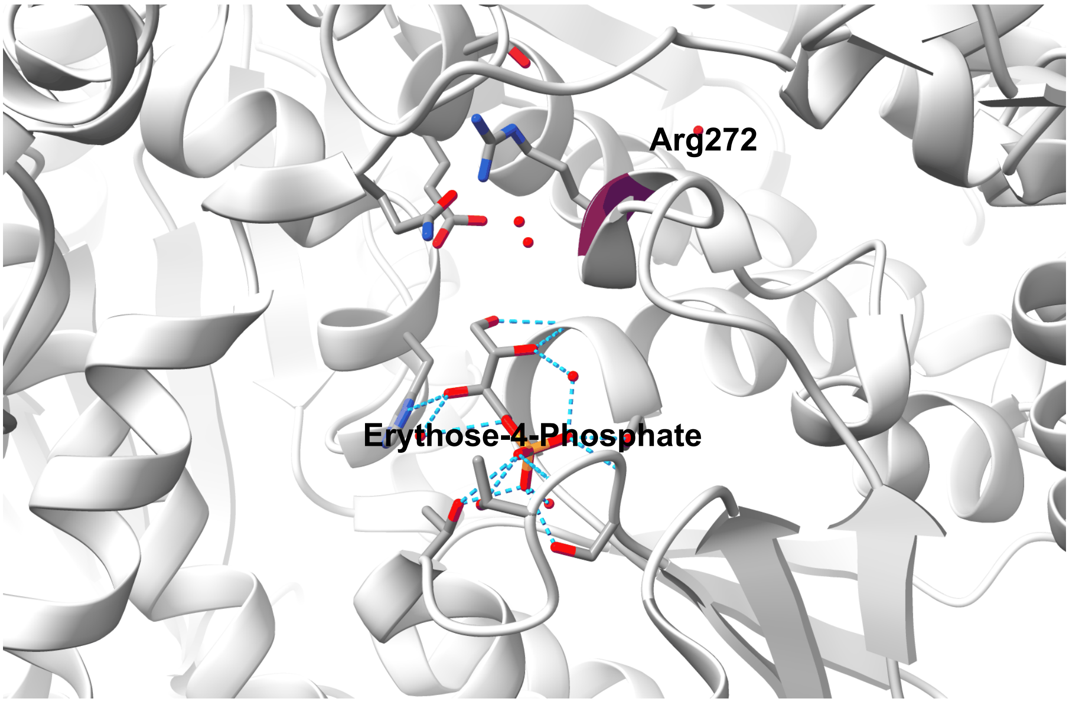

A. Glycolytic substrate of GPI glucose-6-phosphate bound in substrate/active site pocket includes H-bonds to main chain of residue Arg272.

B. An inhibitor of the moonlighting AMF function of GPI, erythose-4-phosphate, does not engage deeper parts of the substrate pocket and has no H-bonds to Arg-272.

This comparison indicates that while introduction of hydrophobic leucine (with FIE R272L) in place of a positively charged arginine would almost certainly disrupt binding of glycolytic substrate and therefore GPI's primary function, it would not necessarily impact the moonlighting cytokine AMF-function. Here we assume AMF inhibitor erythose-4-phosphate binds residues important to the AMF function, which is mediated via binding to AMF-receptor at an interface known to at least partially overlap that of the GPI substrate site.
PDB IDs:1u0f and 1iri on CATH domain 1u0eA02

Supplemental Figure 6 Location of FIEs at oligomeric interfaces of PRDXs

A

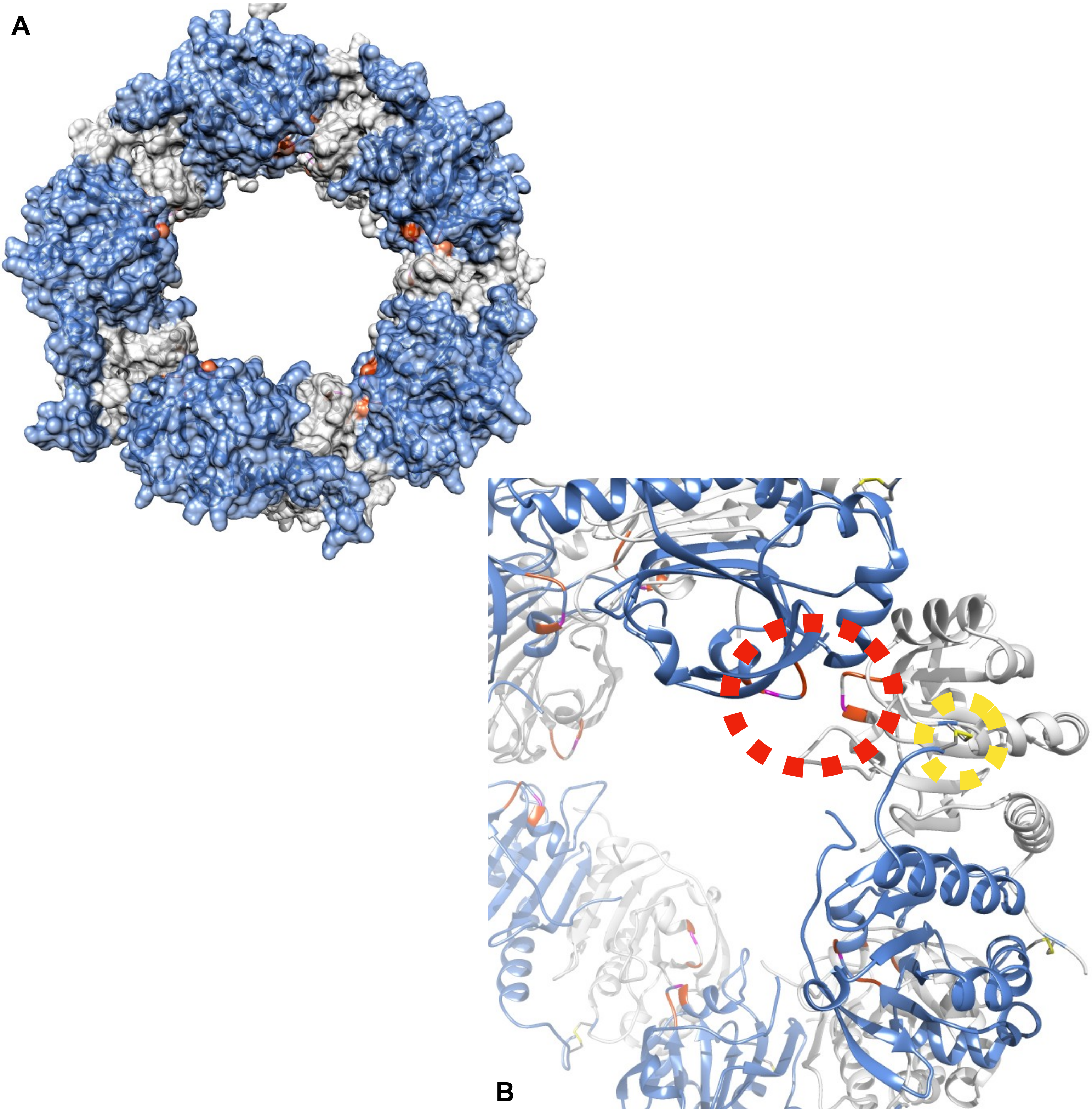

B

A. Higher order decameric structure of PRDXs

2-Cys PRDXs forms obligate homodimers (grey and blue) placing 2 catalytic cysteines (one from each dimer) nearby. Homodimers can form oligomers via a distinct protein interface, as shown here with 5 PRDX2 homodimers forming a decamer.

B. FIEs identified in lung and pan-cancer occur near oligomer interface

All FIEs identified are at oligomeric PRDX interface (*red circle)* which is distinct from the obligate homodimer interface (*yellow circle*) that forms the catalytic site with a cysteine residue from each monomer.

Supplemental Figure 7 Larger images for selected example structures from Figure 4

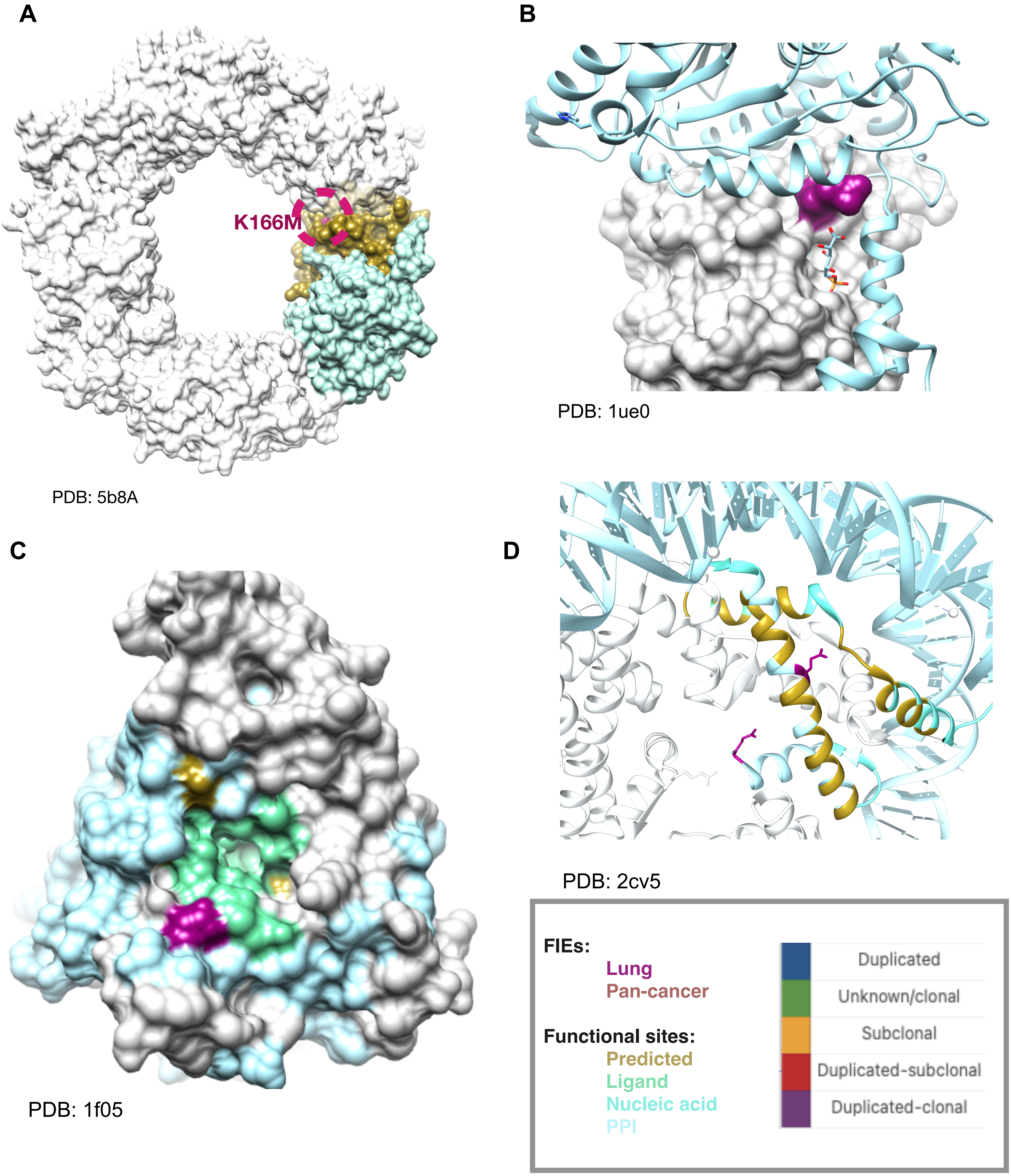

A. Thioredoxin-dependent peroxide reductase *PRDX3*
B. Glucose-6-phosphate isomerase 1 *GPI*
C. Transaldolase *TALDO1*
D. Histone *H3C11*

Supplemental Figure 8 Most diverse CATH superfamilies by number of functional families

A

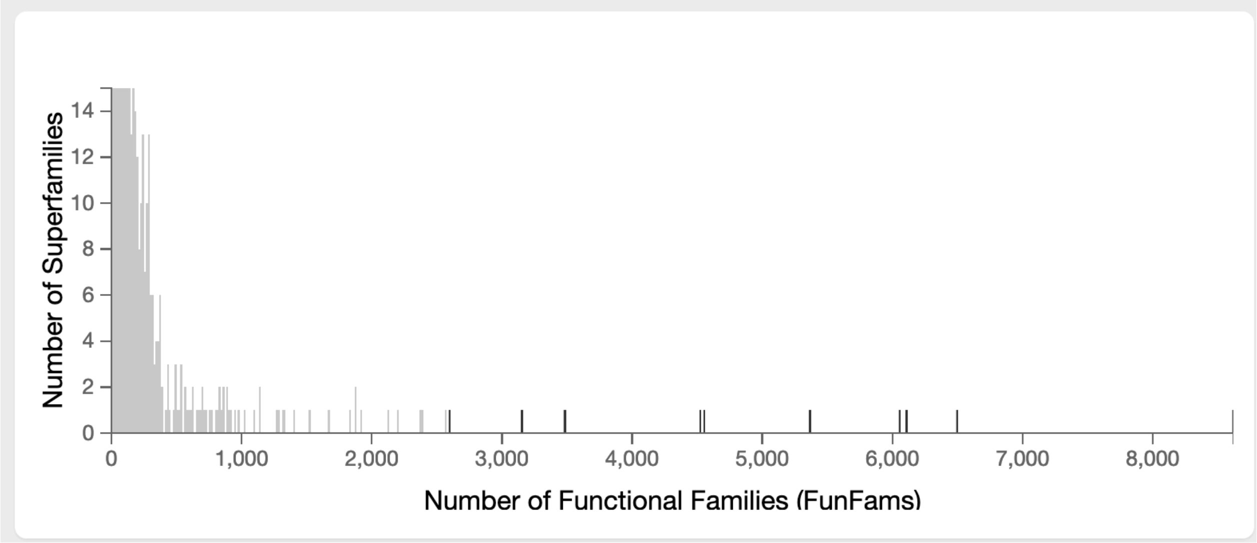

B

Superfamily	Domains	Sequences	Unique EC	Unique GO	Structural Clusters (5A)
3.40.50.300: P-loop containing nucleotide triphosphate hydrolases	9233	5205618	189	6616	158
3.30.160.60: Classic Zinc Finger	359	2184269	8	2462	37
2.60.40.10: Immunoglobulins	31905	2608323	77	4604	94
2.130.10.10: YVTN repeat-like/Quinoprotein amine dehydrogenase	1316	949634	26	3140	25
1.25.40.10: Tetratricopeptide repeat domain	759	1454404	22	2001	41
3.80.10.10: Ribonuclease Inhibitor	709	906325	11	2419	28
1.10.510.10: Transferase(Phosphotransferase) domain 1	7219	962074	36	5880	46
1.25.10.10: Leucine-rich Repeat Variant	694	476232	7	2660	1
3.30.70.330	1527	490198	11	1686	41
3.40.50.720: NAD(P)-binding Rossmann-like Domain	11728	2322073	451	2168	63

A. Histogram of number of superfamilies vs number of functional families. Relatively few superfamilies have very large number (>1000) of functional families

B. Top 10 diverse superfamilies by number of functional families

Supplemental Figure 9 Number of pan-cancer FIEs identified by FunVar score threshold

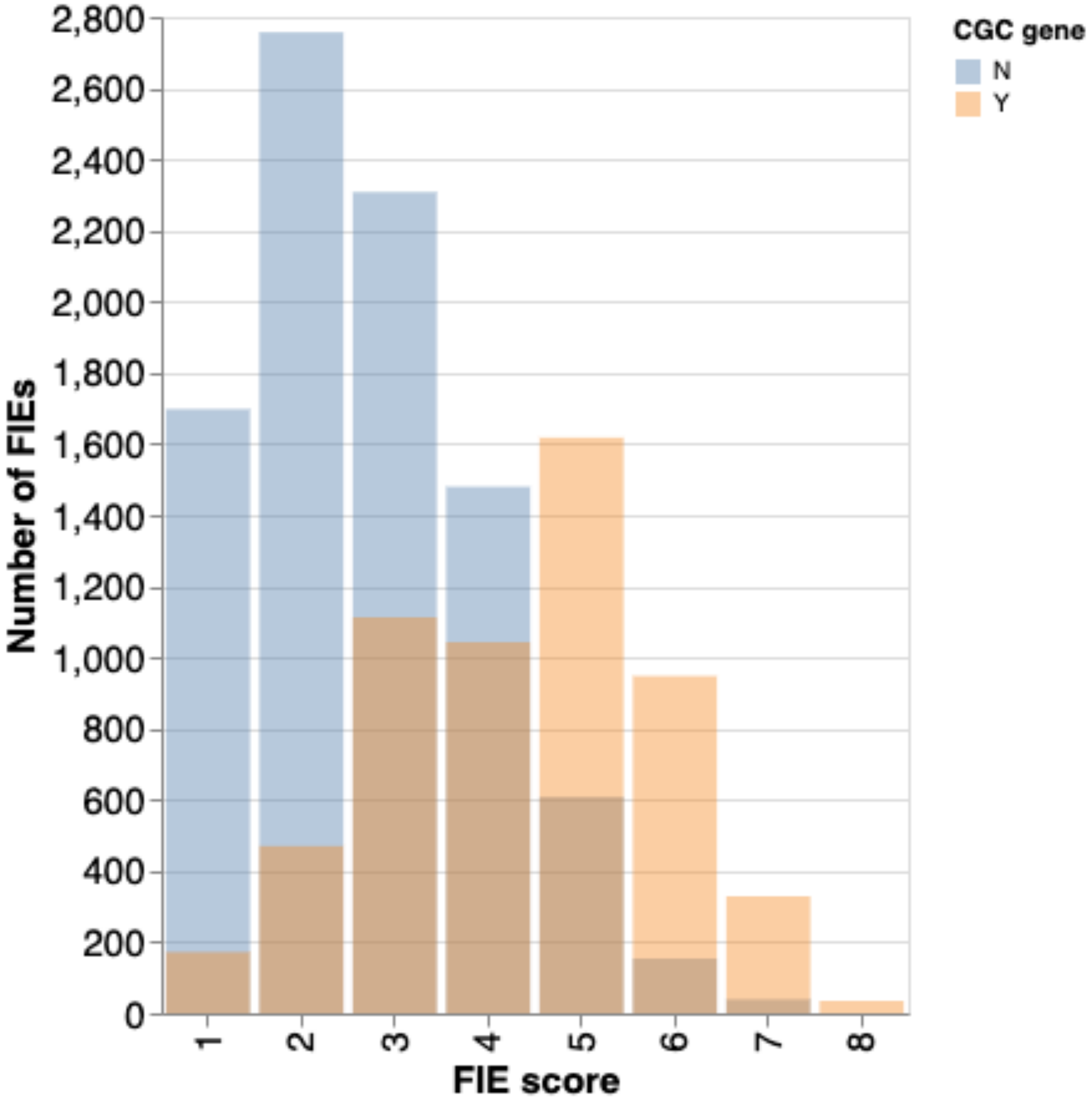

Histogram grouped by FIE-genes being in known cancer gene (yellow) or not (blue). FIEs with a high FunVar score are more likely to be in known cancer genes. For this study, we applied FunVar score threshold at 3, so all FIEs referred to in this manuscript have FunVar scores >=3. As expected, these thresholded FIE-genes are enriched in known cancer genes ($P < 2.2\times10^{-16}$ Chi squared).

Known cancer genes obtained using all genes in Cancer Gene Census.

Supplemental Figure 10 FIEs identified pre- and post-duplication for LUAD/LUSC in TRACERx and TCGA-lung cohorts

TRACERx
LUAD: 239 tumors
LUSC: 134 tumors

	Preduplication FIEs LUAD	Preduplication FIEs LUSC	Postduplication FIEs LUAD	Postduplication FIEs LUSC	Other FIEs* LUAD	Other FIEs* LUSC
**FIEs**	140	51	38	24	86	16
**% of SNVs**	6.4%	3.4%	1.4%	1.8%	2.5%	1.1%
**Genes**	29	18	27	18	44	12
**Tumors**	108	40	33	22	61	12
**FIEs per tumor**	1.30	1.28	1.15	1.09	1.41	1.33

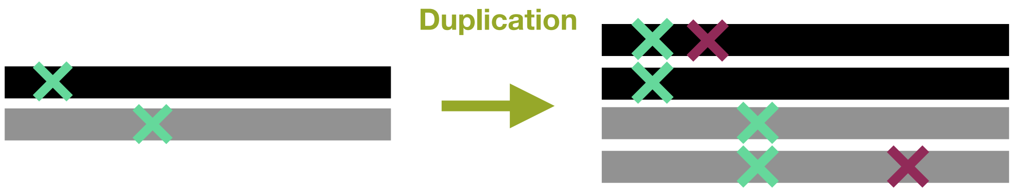

Preduplication FIEs **Postduplication FIEs** **Other FIEs***

TCGA-Lung
LUAD: 387 tumors
LUSC: 342 tumors

	Preduplication FIEs LUAD	Preduplication FIEs LUSC	Postduplication FIEs LUAD	Postduplication FIEs LUSC	Other FIEs* LUAD	Other FIEs* LUSC
**FIEs**	74	57	10	9	128	168
**% of SNVs**	2.6%	2.2%	1.3%	1.7%		
**Genes**	43	35	10	9	75	74
**Tumors**	64	50	10	9	114	137
**FIEs per tumor**	1.16	1.14	1.00	1.00	1.12	1.23

Supplemental Figure 11 Sample coverage estimates for LUAD FIE-gene diversity analysis

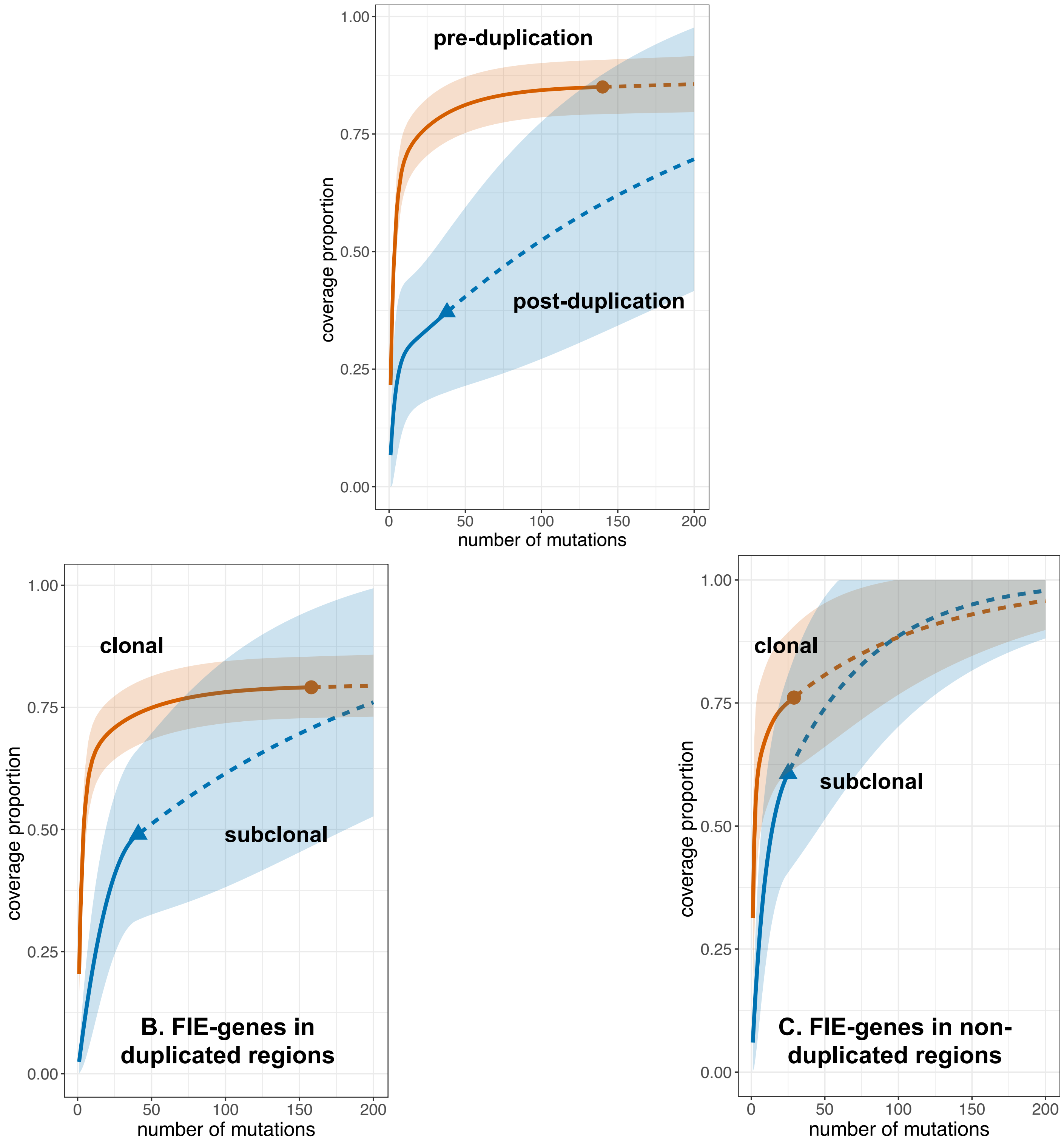

A. Sample completeness plots estimating coverage of LUAD FIE-genes indicate pre-duplication dataset has high coverage (~85%) of all possible FIE-genes, with the completeness curve flattening out. Increasing (for example) the number of tumor samples would be more likely to identify FIEs in genes already captured. Post-duplication, the FIE-gene coverage estimate is lower (<40%) and increasing, indicating that our dataset underestimates the true diversity of post-duplication FIE-genes.

B. In duplicated regions, coverage estimates of clonal and subclonal FIE-genes show similar trends to those for pre- and post-duplication FIE-genes respectively.

C. In non-duplicated regions, low sample sizes result in wide 95%CI for both clonal and subclonal FIE-gene coverage estimates. In addition, both estimates are increasing, meaning true FIE-gene diversity in these classes is not well captured.

Supplemental Figure 12 Datasets used for FunVar-FIE benchmarking

A

Reference dataset	Number of enties	Type	Info
**ClinVar-benign**	**628**	**SNPs (benign)**	2,108 SNPs with clinical significance "Benign"; review status "reviewed by expert panel"; 628 protein coding missense SNVs
**COSMIC cancer mutation census**	**3,984**	**somatic SNVs (cancer drivers)**	Only SNVs marked as clinically significant drivers (3,984 out of 3,637,450 missense SNVs)
**dbSNP**	**9,219**	**SNPs (polymorphisms)**	gnomAD and predicted benign
**TRACERx-drivers**	**594**	**somatic SNVs (cancer drivers - lung)**	Mutations flagged as drivers in NSCLC, based on consensus of predictions and curation as part of TRACERx pipeline

B

Type	Number of *unique* driver mutations	Number of *unique* residue positions	Summary	Filters & remarks
**HotSpot3D**	**2,771**	**1,708**	Predicted driver mutations via 3D clustering and pairwise cluster residue networks	Niu et al 2016
**HotMAPS**		**1,093**	Predicted driver mutations via 3D clustering detecting a higher than expected density of missense mutations	Protein positions lusters (eg EGFR 858), not mutation (eg EGFR L858R)
**3DHotSpots.org**		**1,840**	Predicted driver mutations via 3D clustering within 5A contact residues and permutation testing	Protein positions lusters (eg EGFR 858), not mutation (eg EGFR L858R)
**FunVar-FIE pancan**	**4,734**	**4,032**	Functional Impact Event "driver" mutations via 3D clustering of functional family PDB domains near functional sites	FunVar-score >=3
**FunVar-FIE TRACERx**	**190**	**161**	Functional Impact Event "driver" mutations via 3D clustering of functional family PDB domains near functional sites	**FunVar-score >=3; 355 FIEs**
**TRACERx-drivers**	**410**	**353**	Mutations flagged as drivers in NSCLC, based on consensus of predictions / expert curation as part of TRACERx pipeline	**594 drivers**

A. Reference sets used to define 'Actual Positive" and "Actual Negative" driver mutations

B. Driver prediction tools based on 3D structure

Supplemental Figure 13 FunVar-FIE benchmarking scores compared to cancer driver predictors using 3D protein structures

	FunVar-FIE (pan cancer)		Hotspot3D		HotMAPS		3D Hotspots	
**Classification metric**	**est. mean**	**est. SD**	**est. mean**	**est. SD**	**est. mean**	**est. SD**	**est. mean**	**est. SD**
**accuracy**	**0.579**	0.010	**0.640**	0.013	**0.617**	0.013	**0.610**	0.012
**F1 score**	**0.703**	0.005	**0.735**	0.007	**0.723**	0.007	**0.719**	0.006
**kap**	0.158	0.020	*0.279*	0.026	0.235	*0.025*	0.220	0.023
**mcc**	0.290	0.021	0.399	0.023	0.360	0.024	*0.348*	0.022
**npv**	0.989	*0.015*	0.990	0.011	0.987	0.015	0.991	0.011
**ppv**	0.543	0.006	0.581	0.009	0.567	0.008	0.562	0.007
precision	0.543	0.006	0.581	0.009	0.567	0.008	0.562	0.007
recall	0.998	0.002	0.997	0.003	0.997	0.004	0.998	0.002

FunVar-FIE and other structure-based prediction tools, assessed using missense mutations from TCGA-MC3 pan-cancer and benchmarked against known driver mutations. Drivers are defined as those mutations with evidence for clinical significance in the COSMIC Cancer Mutation Census and predictions use protein residue positions (i.e., cluster sites/residues) for all tools.

Supplemental Figure 14 Summary of predictions from FunVar-FIE (pan-cancer) and other 3D tools on mutations in reference set comprising known cancer driver mutations

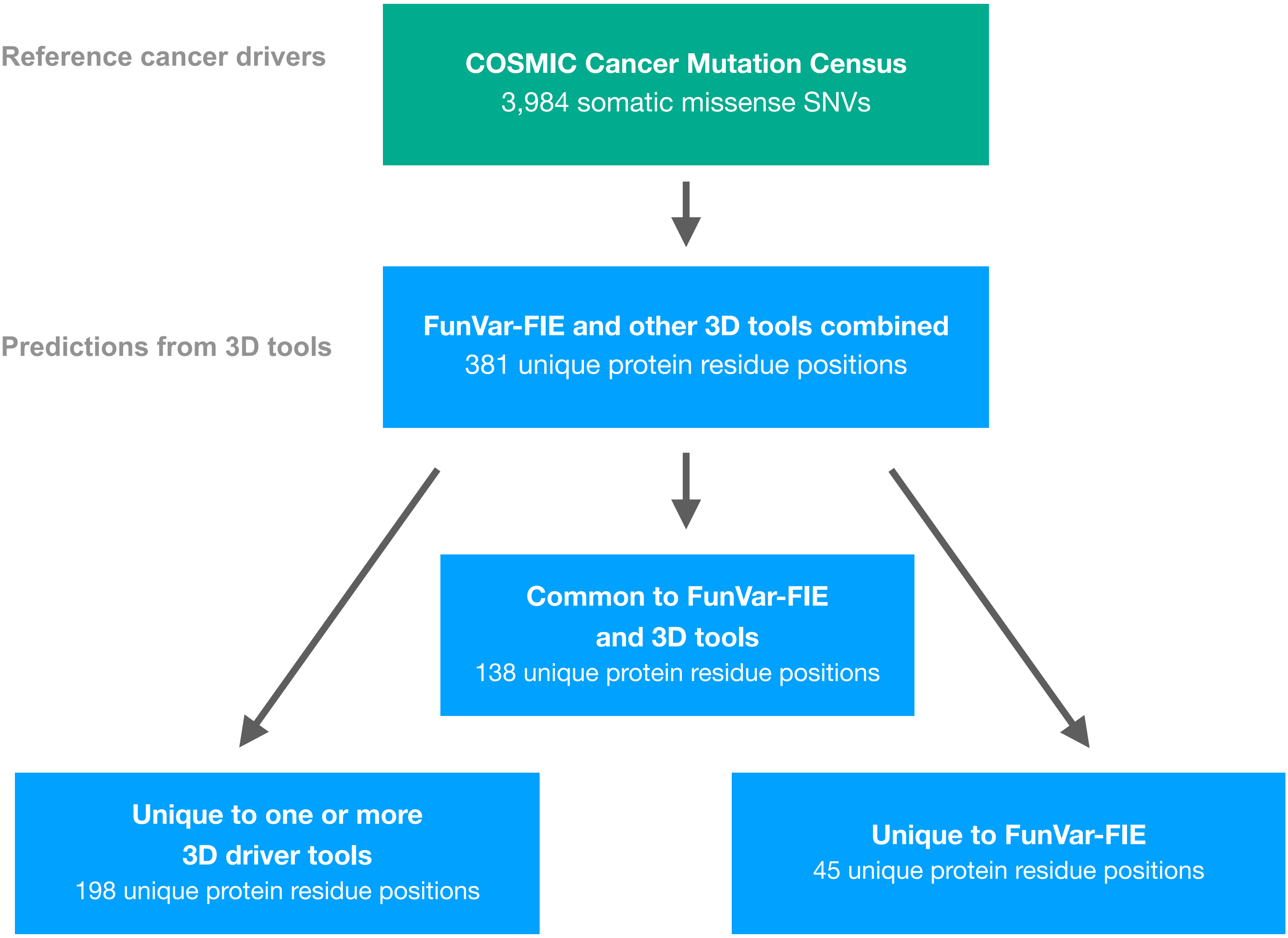

Reason no FIE predicted	Unique residue positions
No functional family or 3D mapped	59
No functional sites	39
Only single functional site nearby	25
**No significant mutation cluster***	69
Other (below FIE score threshold, or only in Tx lung)	6
Total	***198***

***No significant mutation cluster** refers to 60 residue positions, of which 53 in tumor suppressor genes (predominantly *TP53*, *PTEN* and *VHL*), 13 in oncogenes, and 3 in genes with mixed oncogene and tumor suppressor roles.

These FIEs were in 19 functional families, including 7 kinase and 4 metabolic and 3 DNA repair families.

FunVar grouping of mutations by paralogs contributed to identification of FIEs in 8/19 of these families. Paralogs can also be used to transfer functional site annotations from well annotated genes to those with no or limited annotations (see Methods)

These 19 families are given in Supplemental Table 11.

Comparisons use residue number to identify and compare 3D cluster residues identified from FunVar-FIEs, and a consensus score of four other 3D driver tools, with predictions on known cancer drivers from COSMIC CMC.

Supplemental Figure 15 Cancer effect size for pan-cancer functional families with FunVar uniquely predicted drivers in benchmarking

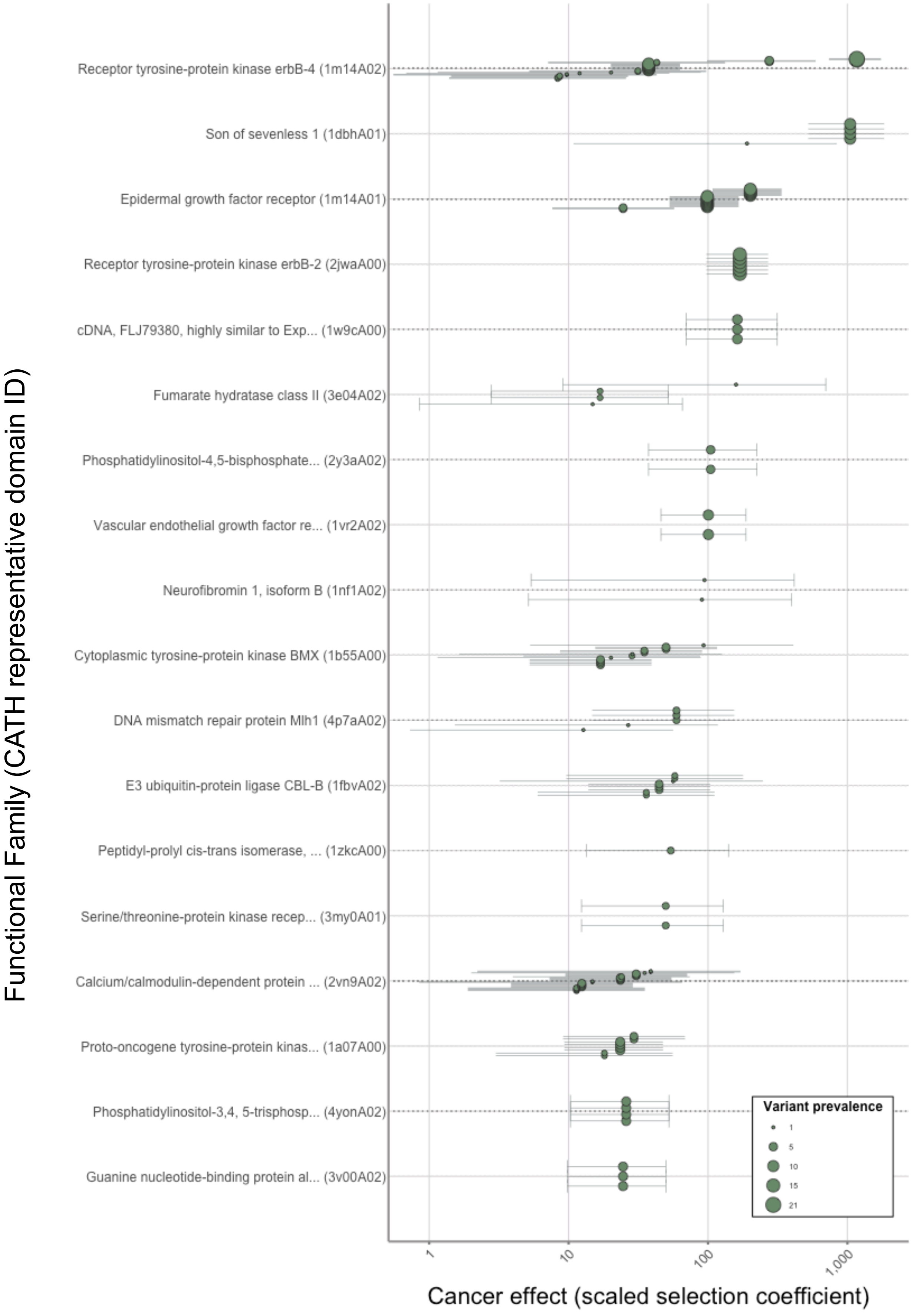

Cancer Effect Size for all variants in functional families with uniquely identified drivers by FunVar in benchmarking Supplemental Table 11. Cancer effects are grouped by functional family, with data points representing cancer effect sizes (95% CI) for mutations in one or more paralogs at distinct alignment positions in the functional family sequence alignment.

Supplemental Figure 16 Cancer effect size for dbSNP benign variants in TCGA pan-cancer, grouped by functional family

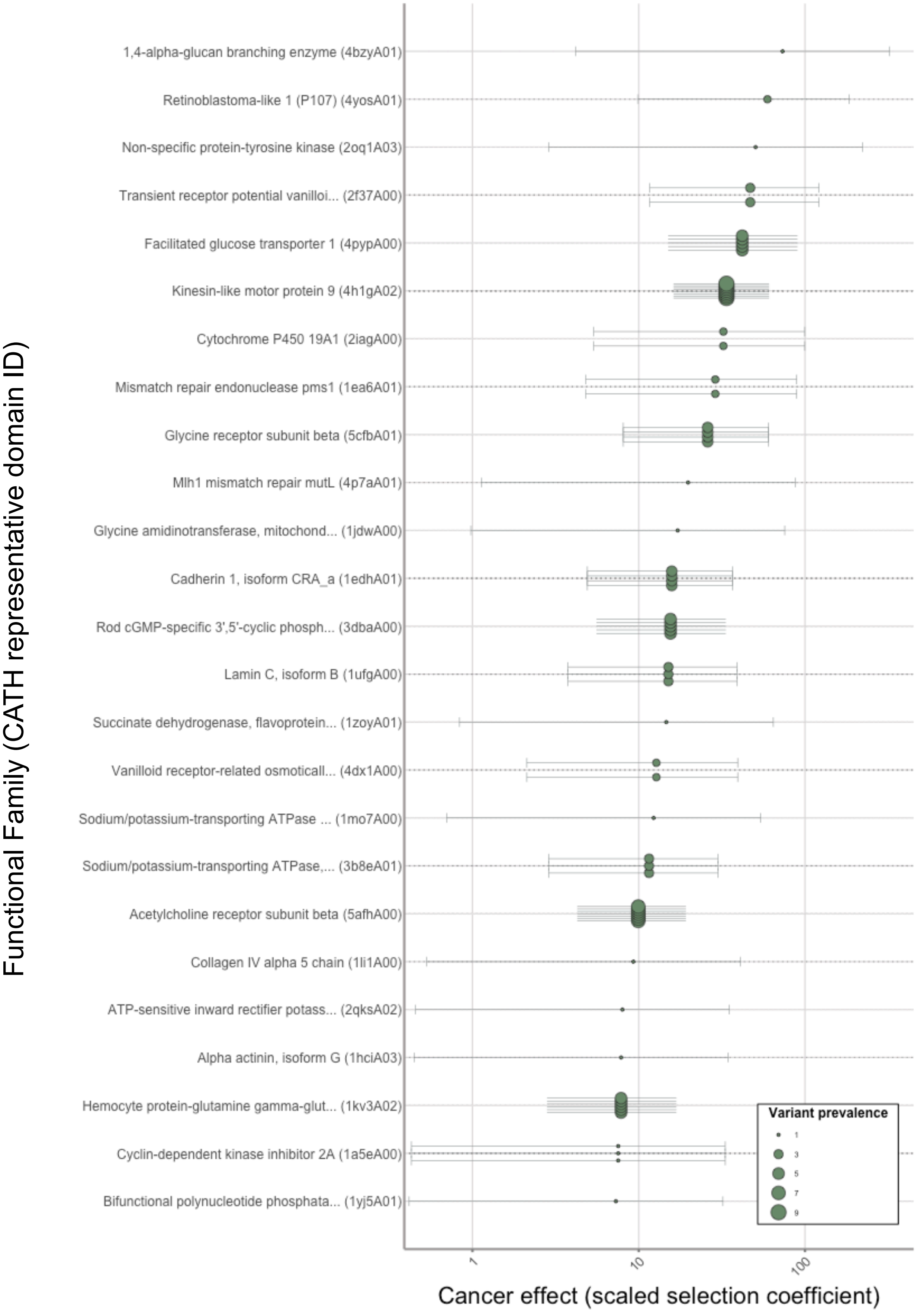

Cancer effect size for dbSNP benign variants in the TCGA pan cancer tumor dataset, grouped by functional family.

Supplemental Figure 17 Cancer effect size for top functionally diverse FIE-containing functional families

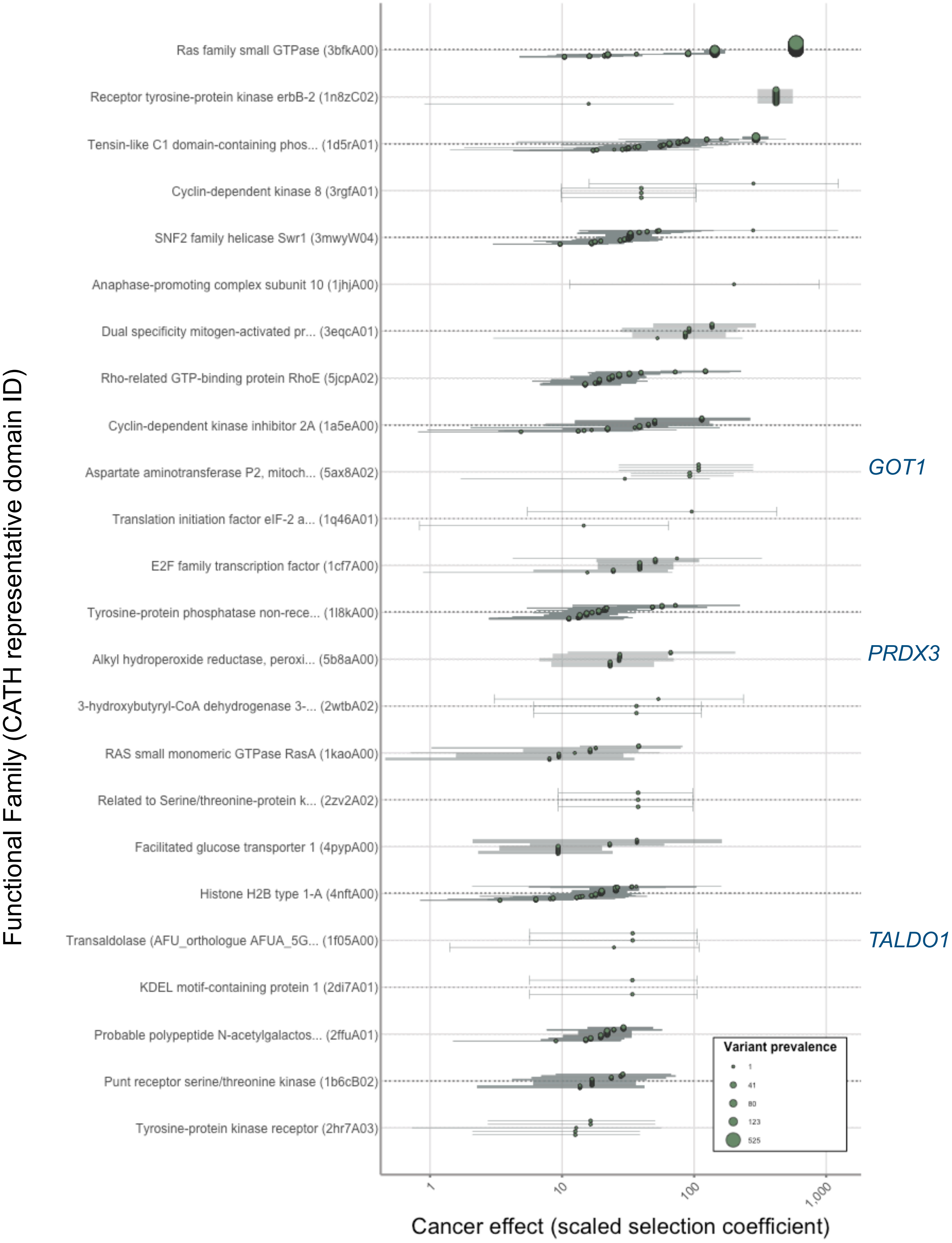

Cancer Effect Size for all variants in functionally diverse families identified by FunVar in Supplemental Table 5. Cancer effects are grouped by functional family, with data points representing cancer effect sizes (95% CI) for mutations in one or more paralogs at distinct alignment positions in the functional family sequence alignment. Selected genes are highlighted from the case studies presented in Figure 4.

Supplemental Figure 18 Cancer effect size for top functionally diverse families in TRACERx LUAD

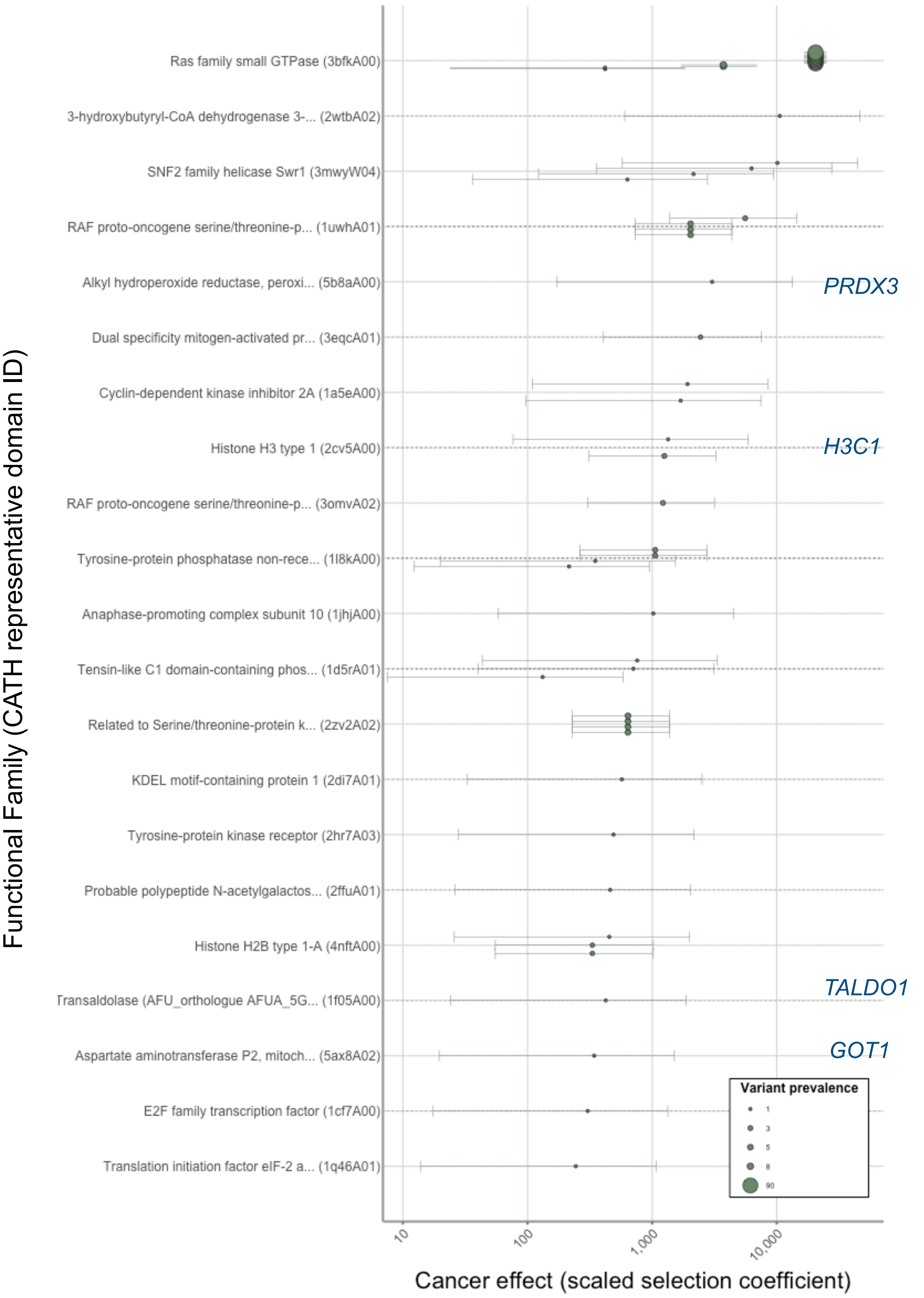

Cancer effect size for TRACERx LUAD for the top functionally diverse families given in Supplemental Table 5. Selected genes are highlighted from the case studies presented in Figure 4.

Supplemental Figure 19 Cancer effect size for top functionally diverse families in TRACERx LUSC

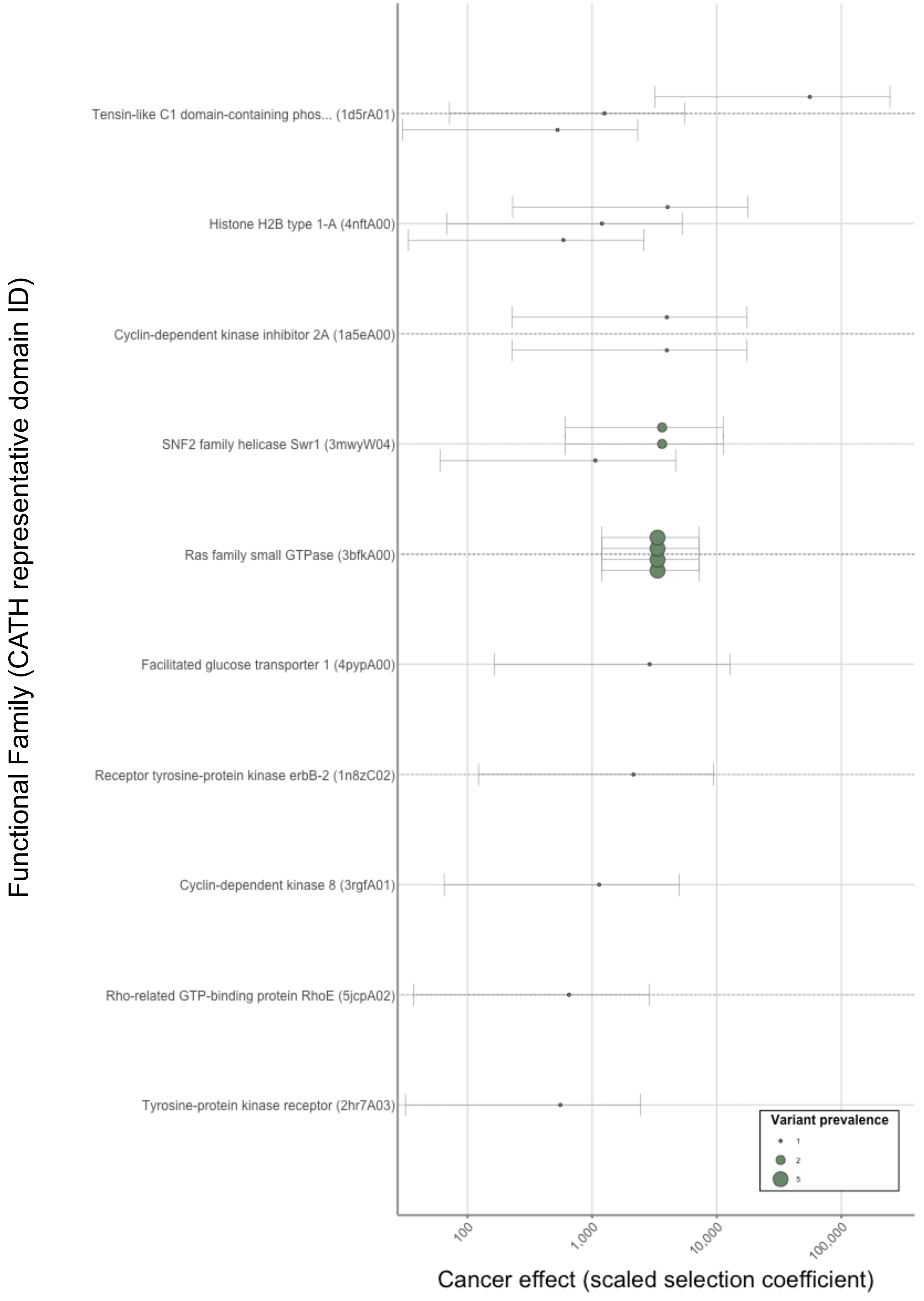

Cancer effect size for TRACERx LUSC for the top functionally diverse families given in Supplemental Table 5.
