# Supplementary figures and images for: Gene duplication is associated with gene diversification and potential neofunctionalization in lung cancer evolution
Source: Genome Res. 2026 Mar;36(3):561–77. doi: 10.1101/gr.278663.123 (PMC12951968; doi:10.1101/gr.278663.123)

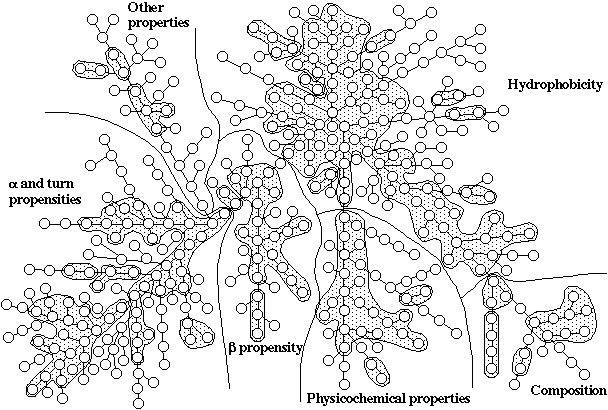

Supplement: Supplement 4 [file Supplemental_Code_1.zip › funvar-tracerx-main/data/aaindex/Fig.4.GIF]

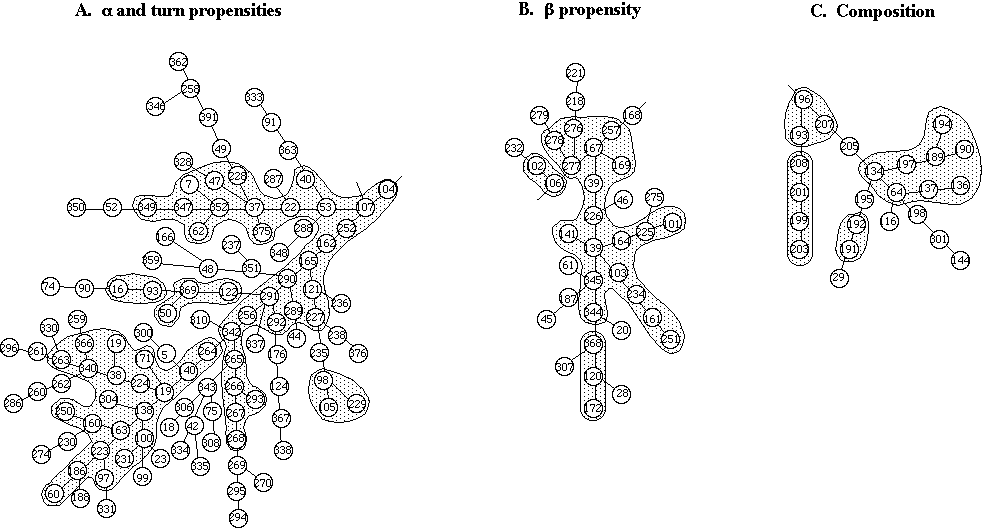

Supplement: Supplement 4 [file Supplemental_Code_1.zip › funvar-tracerx-main/data/aaindex/Fig.5-1.GIF]

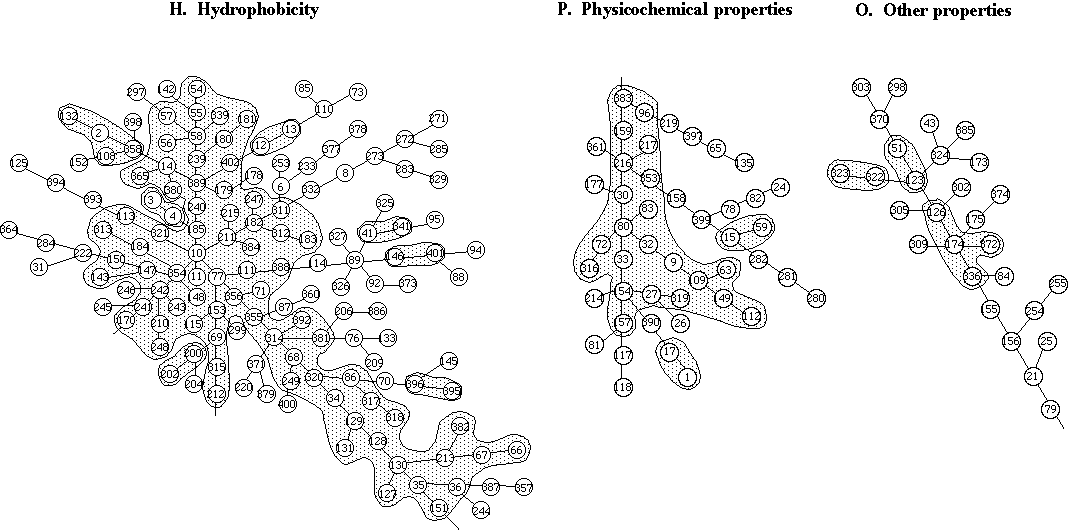

Supplement: Supplement 4 [file Supplemental_Code_1.zip › funvar-tracerx-main/data/aaindex/Fig.5-2.GIF]

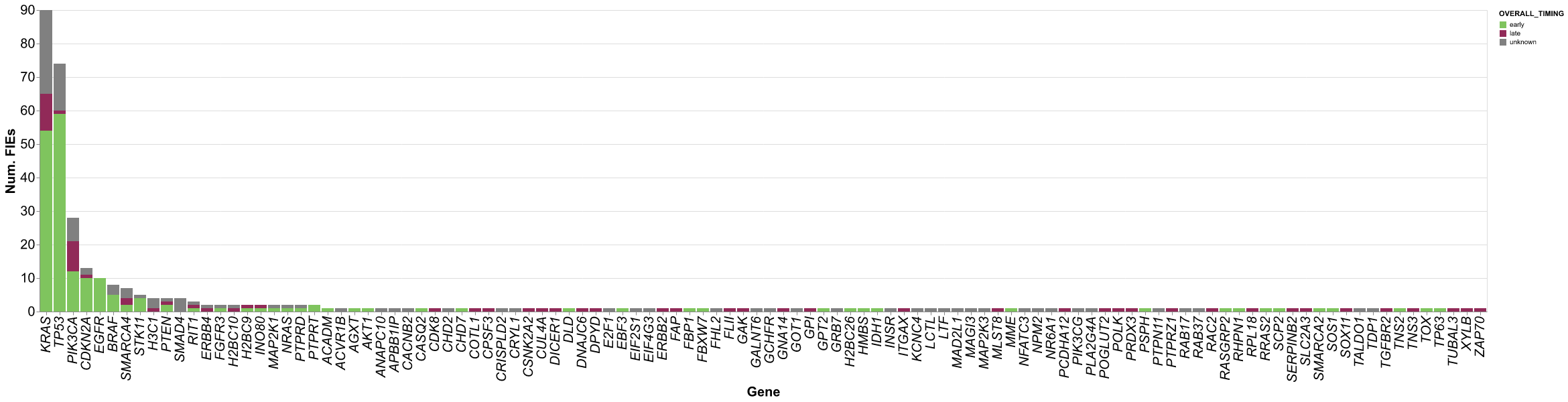

Supplement: Supplement 4 [file Supplemental_Code_1.zip › funvar-tracerx-main/plot/fies_by_gene_all_pre_post_luad_lusc.png]

# LUAD sample coverage – FIE

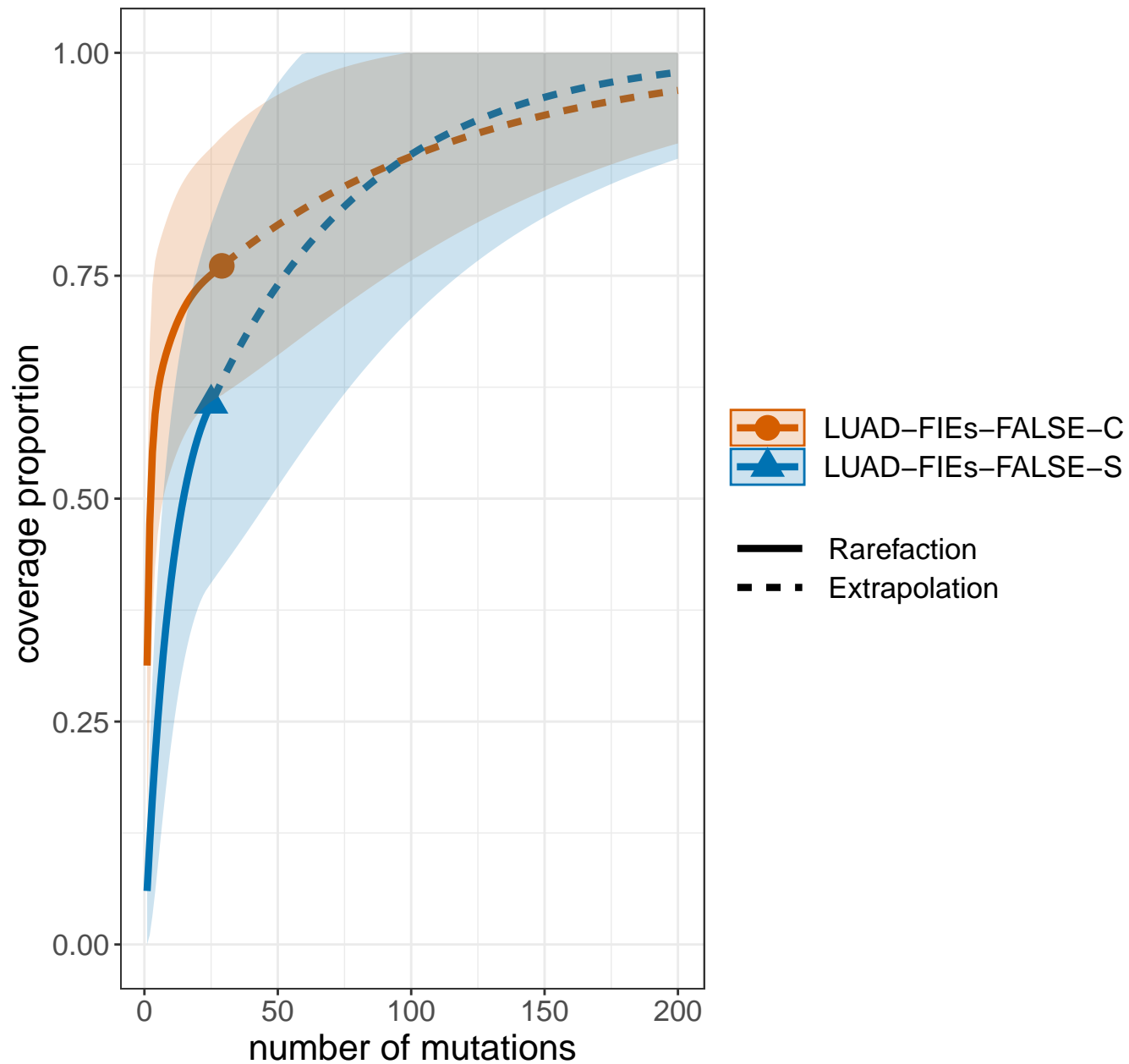

Supplement: Supplement 4 [file Supplemental_Code_1.zip › funvar-tracerx-main/script/diversity_analysis/working/plot_diversity_diversity_yj07f_full_table_gene.csv_duplicated-clonality_duponly_FALSE_q_1_knots_200_nboot_2000_simpler_labels_TRUE_FIE_LUAD_txp_N_conf_0.95_plot_sampcompleteness.pdf]

# LUAD sample coverage – FIE

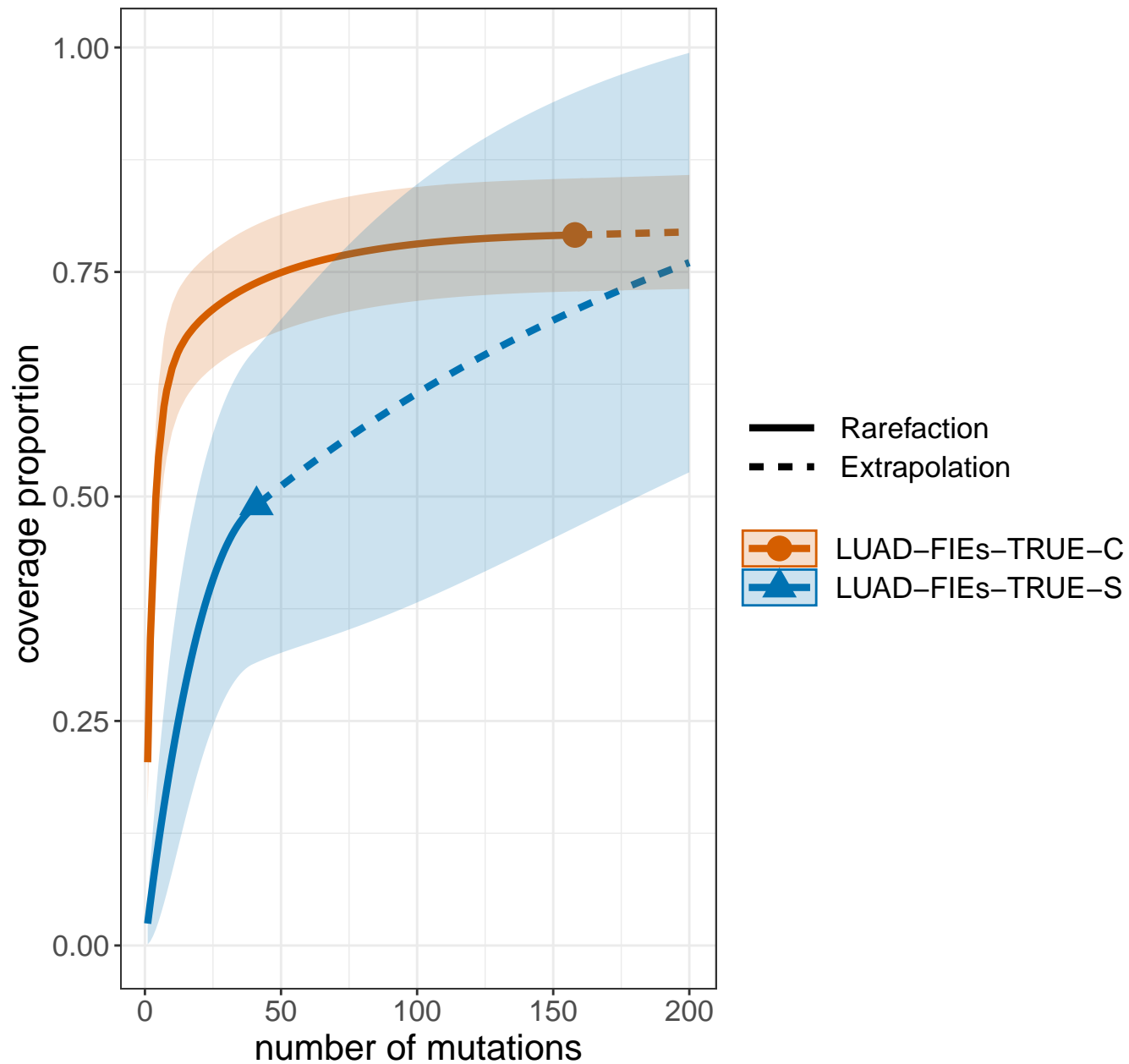

Supplement: Supplement 4 [file Supplemental_Code_1.zip › funvar-tracerx-main/script/diversity_analysis/working/plot_diversity_diversity_yj07f_full_table_gene.csv_duplicated-clonality_duponly_TRUE_q_1_knots_200_nboot_2000_simpler_labels_TRUE_FIE_LUAD_txp_N_conf_0.95_plot_sampcompleteness.pdf]

# LUAD sample coverage – FIE

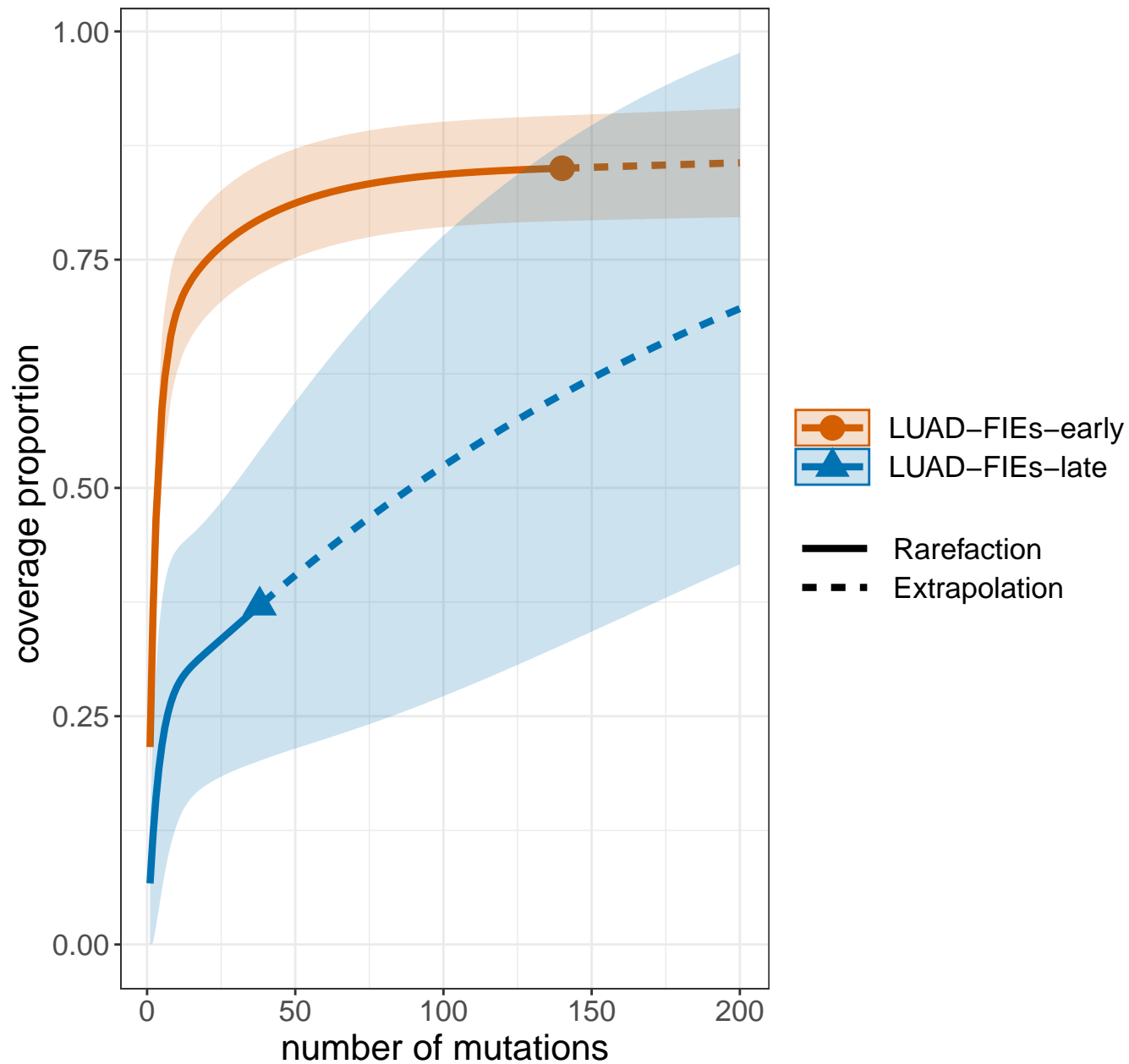

Supplement: Supplement 4 [file Supplemental_Code_1.zip › funvar-tracerx-main/script/diversity_analysis/working/plot_diversity_diversity_yj07f_full_table_gene.csv_timing_q_1_knots_200_nboot_2000_simpler_labels_TRUE_FIE_LUAD_txp_N_conf_0.95_plot_sampcompleteness.pdf]
